# Mechanometabolism of cell adhesion: Vinculin regulates bioenergetics via RhoA-ROCK

**DOI:** 10.1083/jcb.202504025

**Published:** 2026-01-20

**Authors:** Emily D. Fabiano, Elle P. Techasiriwan, Lindsey N. Sabo, Nathaniel Seluga, Brenton D. Hoffman, Cynthia A. Reinhart-King

**Affiliations:** 1 Department of Biomedical Engineering, Vanderbilt University, Nashville, TN, USA; 2 Department of Bioengineering, Rice University, Houston, TX, USA; 3 Hopkins Authentic Research Program in Science, Hopkins School, New Haven, CT, USA; 4 Department of Biomedical Engineering, Duke University, Durham, NC, USA

## Abstract

Cell migration and cytoskeletal remodeling are energetically demanding processes. Reorganizing the cytoskeleton requires ATP to fuel the actomyosin complex, enabling cells to adhere to and migrate through a matrix. While it is known that energy is required for cell migration, the mechanism by which cell–extracellular matrix adhesion influences cell energetics is unclear. Here, we investigated the relationship between cell–extracellular matrix adhesion and cellular metabolic state with a focus on vinculin given its role in connecting the cytoskeleton to focal adhesions and extracellular space. Knocking out vinculin increases the metabolic activity in cells and results in fast, frequent Rho kinase activity–dependent changes in cell shape and protrusions. The cellular protrusion dynamics and bioenergetics are interrelated processes, as stimulating RhoA/Rho kinase activity increases dynamic blebbing protrusions and energy production, and inhibiting metabolism decreases the frequency of blebbing cell protrusions. This link between cell–extracellular matrix adhesion and bioenergetics provides a novel basis by which cellular metabolism and cell migration could be controlled.

## Teaser

The focal adhesion protein vinculin affects the dynamics of the actin cytoskeleton, and reorganization of the actin cytoskeleton is an energetically demanding process important in cell migration. Here, Fabiano et al. establish a relationship between vinculin, cell bioenergetics, and migration behavior, and the RhoA/ROCK/myosin II pathway in MDA-MB-231 cells.

## Introduction

Many pathological and physiological processes such as wound healing, angiogenesis, and tumor metastasis require cellular energy to fuel cell migration (Liu et al., 2022; Daniel et al., 1986). Migration requires coordinated actin cytoskeleton remodeling, an energy-intensive process (Bernstein and Bamburg, 2003; Wu et al., 2021; Daniel et al., 1986). Actin cytoskeleton remodeling typically supports cell migration by mediating the formation and retraction of protrusions, generating traction stresses, and deforming the cell body to move through complex extracellular matrix (ECM) architecture (Gardel et al., 2008; Datta et al., 2021; Li et al., 2019; Zanotelli et al., 2018; Pasqualini et al., 2018; Mason and Martin, 2011; Wolf et al., 2013). During cell migration, actin flow is coupled to the ECM through focal adhesions (FAs), and generation of rearward tractions and protrusion of the leading edge aid in propelling the cell forward (Swaminathan and Waterman, 2016). Since remodeling the actin cytoskeleton consumes large amounts of energy in cells but is also a key player in cell migration processes, previous work has probed the role of the actin cytoskeleton as a link between cell migration and bioenergetics (Bernstein and Bamburg, 2003; Cunniff et al., 2016; Hu et al., 2016; Park et al., 2020; Tojkander et al., 2018; Wu et al., 2021). Energy demands tend to increase with substrate stiffness or cell confinement (Zanotelli et al., 2019; Wu et al., 2021; Park et al., 2020; Xie et al., 2021). Greater energy demands are often associated with membrane remodeling, including increased protrusive activity or increased formation of F-actin bundles in cells, and can be induced by changes in the mechanical properties of the extracellular environment, as alterations to substrate properties typically affect cell–ECM adhesion (Bangasser et al., 2017; Tervonen et al., 2023). However, the molecular mechanisms at cell–ECM adhesions that mediate actin cytoskeleton-linked changes in bioenergetics are not clear.

FA protein vinculin is a critical component in modulating connections between F-actin and ECM-bound integrins, mediating force transmission into and out of the cell by directly binding to F-actin and to other FA proteins. Since vinculin stabilizes the connection between the actin cytoskeleton and the ECM, vinculin engagement with actin also slows down the retrograde flow rate of actin (Thievessen et al., 2013; Jannie et al., 2015). Vinculin is known to interact with the metabolic machinery of the cell (Muhs et al., 1997; Tan et al., 2014; Wang, 1986). Metabolic inhibition causes vinculin to delocalize from adhesions (Muhs et al., 1997; Wang, 1986), while stimulation with ATP is known to transiently promote punctate vinculin clustering in the cytosol, likely resulting from direct activation of membrane ATP receptors (Tan et al., 2014). Although studies have demonstrated that vinculin and cell bioenergetics both play substantial roles in determining cell migration behavior and the energetic state of the cell can affect vinculin dynamics, the reciprocal relationship is not clear: specifically, it is not known what role vinculin has in determining the metabolic state of a cell.

Here, we investigated how vinculin mediates the bioenergetic phenotype of cells and how the changes in bioenergetics upon vinculin manipulation affected migration behavior utilizing MDA-MB-231 (MDA) vinculin-knockout (Vcl KO) cells and probes of cell energetics. Interestingly, without vinculin, cellular energy utilization and production increased. While the increased bioenergetics induced by vinculin disruption did not fuel faster cell migration or more proliferation, it was associated with more dynamic cell protrusion and shape-changing behavior. Pharmacological inhibition of Rho kinase (ROCK) and myosin II using Y-27632 and blebbistatin (Blebb), respectively, prevented the dynamic changes in cell shape and bioenergetic phenotypes of the cells that were induced by increased RhoA activity as a result of knocking out vinculin. Accordingly, stimulating Rho activity in parental MDA cells induced dynamic cell shape changes and increased energy production. Together, our results suggest that cellular protrusion dynamics are energy-intensive, and vinculin plays a key role in suppressing protrusive activity and a concordant increase in metabolic activity.

## Results and discussion

### Vinculin disruption increases bioenergetics of MDA-MB-231 cells

**Figure 1.**
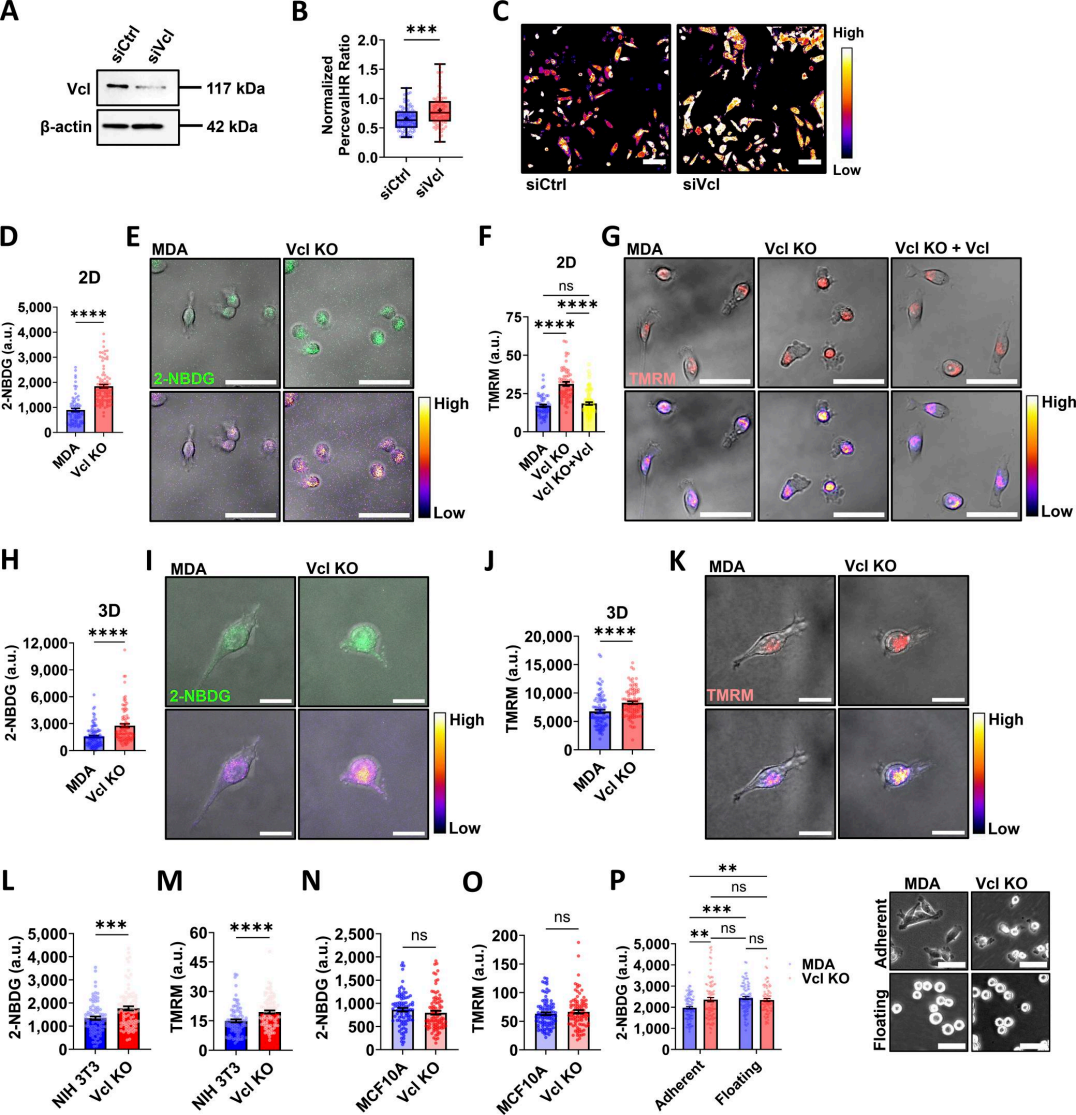
**Vinculin depletion increases bioenergetics of MDA-MB-231 cells. (A)** Representative western blot of MDA-MB-231 cells expressing PercevalHR and pHRed probes transfected with siRNA to knock down vinculin (siVcl), including a scrambled control (siCtrl). **(B)** Quantification of the PercevalHR ratiometric signal for MDA cells expressing PercevalHR and pHRed probes treated with siRNA to knock down vinculin, including a scrambled control (siCtrl) and siVcl (*N* = 3, *n* = 91–92 cells). **(C)** Representative ratiometric heatmaps of siCtrl and siVcl MDA cells expressing PercevalHR and pHRed. Scale bar, 100 µm. **(D)** Mean intensity of 2-NBDG of cells on 2D glass surfaces (*N* = 3, *n* = 70–89 cells). **(E)** Representative images of 2-NBDG staining displayed using a fluorescent channel (top) and the Fire lookup table (bottom) to indicate the relative intensity of the fluorescent signal. Scale bar, 50 µm. **(F)** Mean intensity of TMRM of cells on 2D glass surfaces (*N* = 3, *n* = 58–84 cells). **(G)** Representative images of TMRM staining displayed using a fluorescent channel (top) and the Fire lookup table (bottom) to indicate the relative intensity of the fluorescent signal. Scale bar, 50 µm. **(H)** Mean intensity of 2-NBDG for cells embedded in a 1.5 mg/ml collagen matrix (*N* = 3, *n* = 91–103 cells). **(I)** Representative images of 2-NBDG staining in a 1.5 mg/ml collagen matrix displayed using a fluorescent channel (top) and the Fire lookup table (bottom) to indicate the relative intensity of the fluorescent signal. Scale bar, 20 µm. **(J)** Mean intensity of TMRM for cells embedded in a 1.5 mg/ml collagen matrix (*N* = 3, *n* = 91 cells). **(K)** Representative images of TMRM staining in a 1.5 mg/ml collagen matrix displayed using a fluorescent channel (top) and the Fire lookup table (bottom) to indicate the relative intensity of the fluorescent signal. Scale bar, 20 µm. **(L)** Mean intensity of 2-NBDG in NIH/3T3 cells on 2D glass surfaces (*N* = 3, *n* = 89–91 cells). **(M)** Mean intensity of TMRM in NIH/3T3 cells on 2D glass surfaces (*N* = 3, *n* = 90–94 cells). **(N)** Mean intensity of 2-NBDG in MCF10A cells on 2D glass surfaces (*N* = 3, *n* = 88–90 cells). **(O)** Mean intensity of TMRM in MCF10A cells on 2D glass surfaces (*N* = 3, *n* = 90–96 cells). **(P)** Mean intensity of 2-NBDG for MDA (*N* = 3, *n* = 93–98 cells) or Vcl KO (*N* = 3, *n* = 79–86 cells) cells grown either on a tissue culture–treated surface (Adherent) or in a nonadherent plate (Floating), including representative phase-contrast images of MDA or Vcl KO cells seeded on a tissue culture surface or suspended in an ultra-low attachment plate. Scale bar, 50 µm. The box-and-whisker plot shows median and 25th/75th percentile (box), min to max (whiskers), and mean (+). Bar graphs denote the mean ± SEM. ns = not significant, **P < 0.01, ***P < 0.001, ****P < 0.0001. siVcl, siRNA targeting vinculin. Source data are available for this figure: SourceData F1.

**Figure S1.**
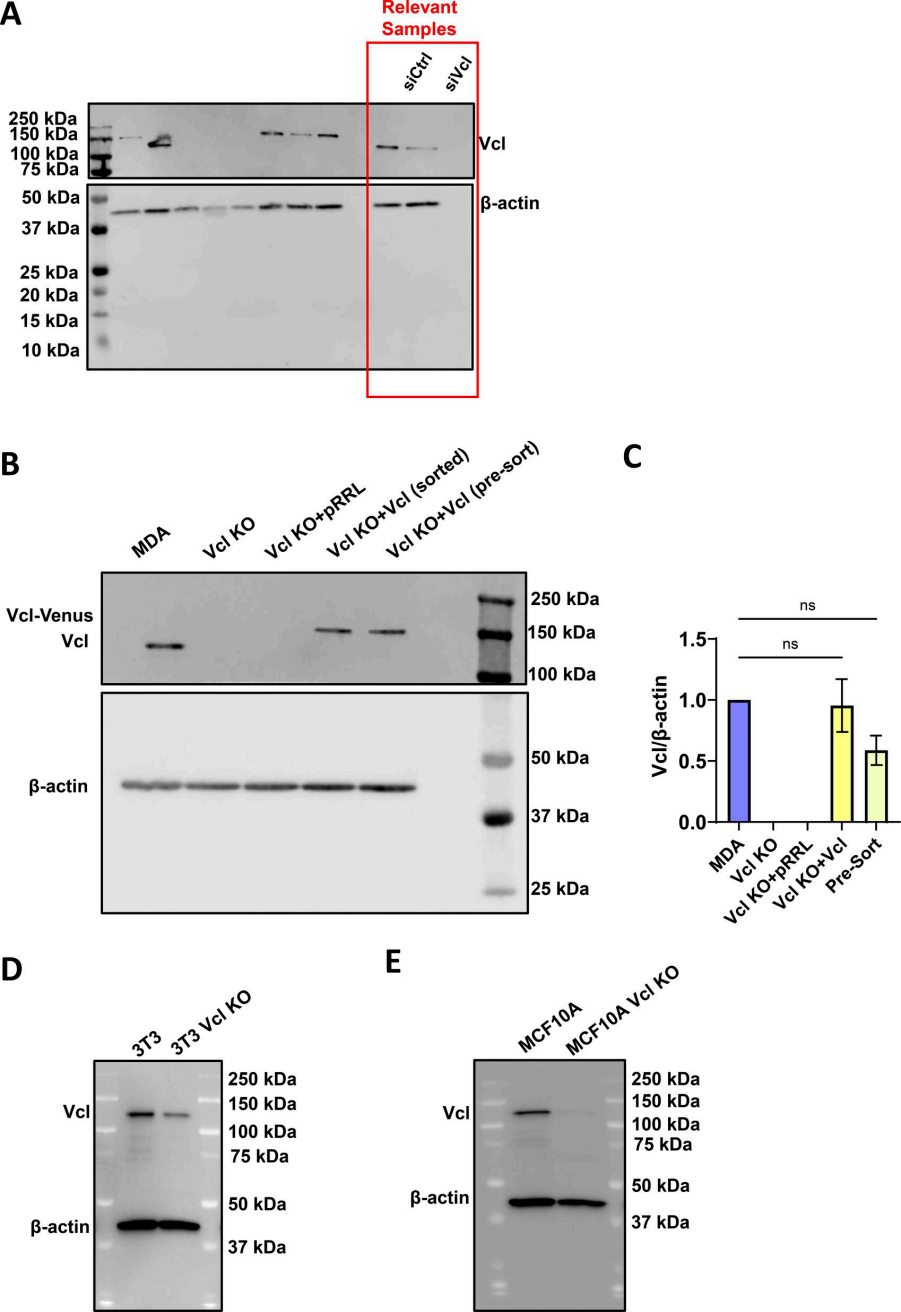
**Representative western blots. (A)** Representative western blot confirming Vcl knockdown in MDA-MB-231 cells expressing PercevalHR and pHRed probes, including a scrambled control (siCtrl) and siVcl. Relevant samples are outlined with a red box, and uncropped membrane is included to show the ladder. **(B and C)** Representative western blot and (C) quantification from three independent western blots confirming Vcl KO and rescue in MDA-MB-231 cells (*N* = 3). The Pre-Sort population refers to the Vcl KO cells transduced with Vcl-Venus before positively expressing Venus cells were sorted using FACS. Bar graph denotes the mean ± SEM. ns = not significant. **(D)** Representative western blot to confirm Vcl KO in NIH/3T3 cells. **(E)** Representative western blot to confirm Vcl KO in MCF10A cells. siVcl, siRNA targeting vinculin; FACS, fluorescence-activated cell sorting. Source data are available for this figure: SourceData FS1.

Cell migration is an energetically demanding process, as it requires reorganization of the cytoskeleton (Wu et al., 2021; Datta et al., 2021). However, the coordination between migration and energetics is not clear. In our previous work, MDA cells lost their ability to polarize active mitochondria to the front of the cell during migration through collagen channels when vinculin was knocked out (Mosier et al., 2023). Prior work suggests that without vinculin, cells move more quickly in both two dimensions (2D) and three dimensions (3D) (Gao et al., 2017; Thievessen et al., 2013; Thievessen et al., 2015). Taking these findings together, it is thought that vinculin may play a role in regulating cell bioenergetics. To investigate the relationship between vinculin and bioenergetics, we performed a partial knock down of vinculin in MDA cells (Fig. 1 A and Fig. S1 A), and cell energetic level was measured using PercevalHR, a live reporter of the ATP:ADP ratio (Tantama et al., 2013). The PercevalHR ratiometric signal was greater in vinculin knockdown cells compared to cells transfected with the scrambled control (Fig. 1, B and C), suggesting that the vinculin knockdown cells have increased energy utilization.

**Figure S2.**
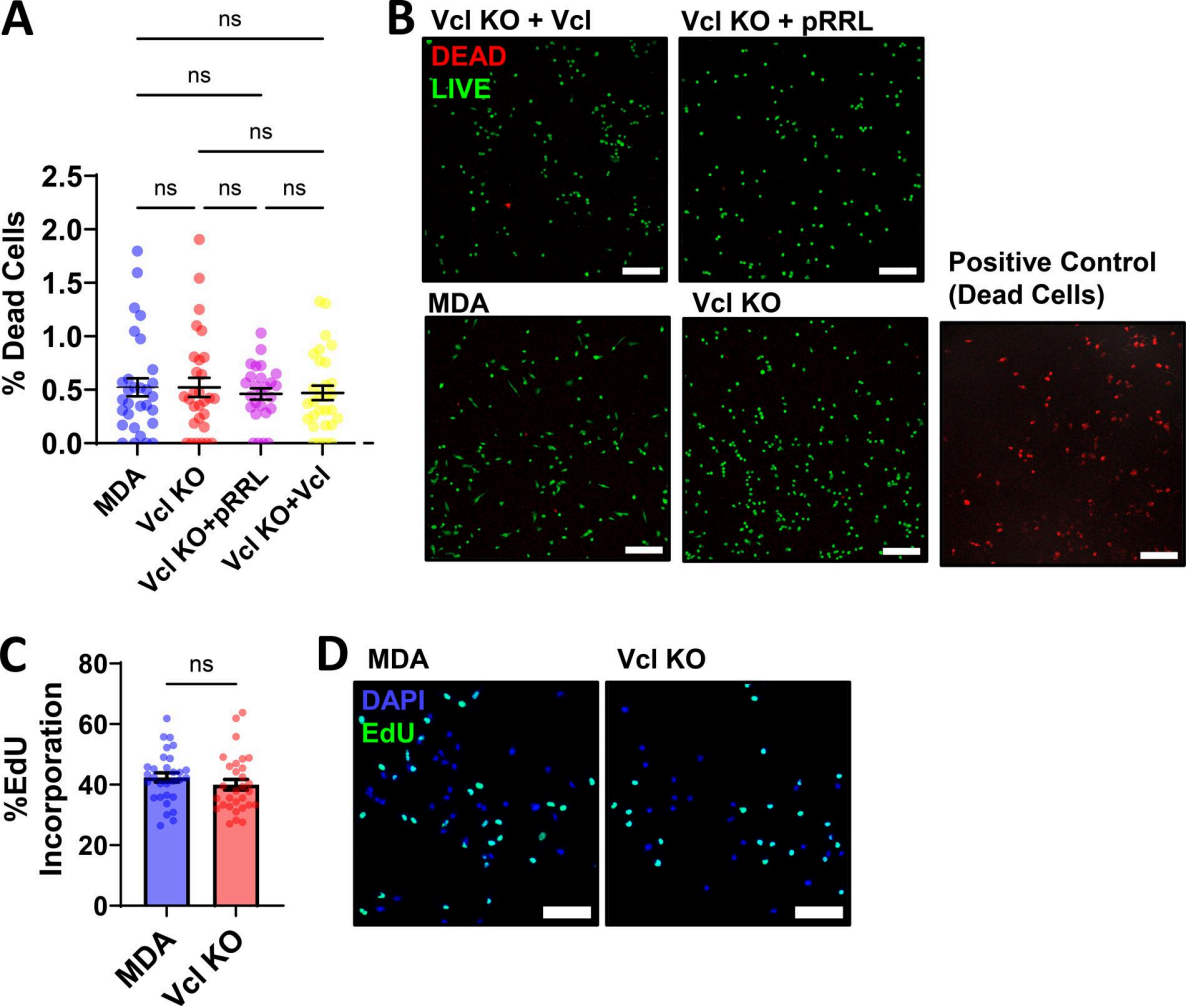
**Characterization of the MDA-MB-231 Vcl KO cell line. (A)** Quantification of the percentage of dead cells from 30 fields of view per condition using a LIVE/DEAD kit (*N* = 3). **(B)** Representative images of cell lines indicated treated with a LIVE/DEAD kit to assess cell viability in 2D. The right panel represents the positive control condition. Scale bar, 200 µm. **(C and D)** Percentage of EdU incorporation in parental MDA-MB-231 and Vcl KO cells and (D) representative images of EdU incorporation and DAPI to assess cell proliferation (*N* = 3). Scale bar, 100 µm. Graphs denote the mean ± SEM. ns = not significant.

To better understand the role vinculin may play in regulating cell bioenergetics, we used CRISPR/Cas9 to generate a Vcl KO cell line in MDA cells, followed by generation of a vinculin rescue cell line by expressing vinculin-Venus in the Vcl KO cells (Vcl KO+Vcl) (Fig. S1, B and C). Cell viability and proliferation were not affected by knocking out vinculin (Fig. S2, A–D). Since vinculin knockdown results in an increase in ATP:ADP ratio (Fig. 1, B and C), we hypothesized that cells enhance glucose uptake to meet ATP demands. Consistent with the results using the PercevalHR probe, glucose uptake was greater in the Vcl KO cells compared with the parental MDA cells on 2D surfaces (Fig. 1, D and E). For 2-NBDG experiments, the Vcl KO+Vcl cells were not used due to the optical overlap of the Venus tag with 2-NBDG signal. Since oxidative phosphorylation may also be a major ATP-producing pathway in cells, we assessed mitochondrial membrane potential using TMRM, and found this metric was also increased in the Vcl KO cells in 2D (Fig. 1, F and G) (Wu et al., 2021; Zheng, 2012; Bonuccelli et al., 2010; Davis et al., 2020). We extended our investigation into 3D by embedding cells in a collagen matrix to recapitulate a more physiologically relevant environment. Similar to our findings in 2D, the Vcl KO cells increased glucose uptake (Fig. 1, H and I) and mitochondrial membrane potential when embedded in collagen (Fig. 1, J and K) compared with control cells. Together, these data suggest that the Vcl KO cells have increased energy production that is maintained independent of the environment in which they are migrating.

To determine whether our findings are generalizable to other cell types, we knocked out vinculin in NIH/3T3 embryonic fibroblasts and MCF10A nonmalignant mammary cells and assessed glucose uptake and mitochondrial membrane potential (Fig. S1, D and E). NIH/3T3 cells were chosen as a commonly used migratory, mesenchymal fibroblast cell line, while MCF10A cells were selected as a nonmalignant breast epithelial cell line for their relevance in studying the role of vinculin in both cell–cell and cell–ECM adhesions. While the NIH/3T3 cells exhibit increased bioenergetics upon Vcl KO, the MCF10A bioenergetics remains unchanged when cultured as islands (Fig. 1, L–O). Like the MDAs, NIH/3T3 cells are considered a migratory cell line, thus requiring energy to support migration-related processes. However, MCF10A cells are less migratory epithelial cells, and the energetic requirements in epithelial cells often focus on the importance of ATP in maintaining cell–cell adhesion (Cavanaugh et al., 2001; Canfield et al., 1991; JanssenDuijghuijsen et al., 2017). Together, our results suggest that vinculin-mediated bioenergetic phenotypes may be unique to migratory cell lines.

To determine whether the energetic phenotypes observed are specific to vinculin-mediated mechanosensing, we compared glucose uptake for vinculin-expressing MDA cells cultured on a tissue culture–treated surface, which promotes adhesion, or cultured in a nonadherent environment, in which the cells are floating. Interestingly, the floating MDA cells exhibited greater glucose uptake compared with the MDA cells grown on tissue culture–treated surfaces (Fig. 1 P), suggesting that loss of cell–ECM adhesion could be associated with increased glucose uptake. The glucose uptake by the Vcl KO cells does not change in response to the loss of cell–ECM adhesion (Fig. 1 P), suggesting that either the energetics are already saturated or vinculin deletion induces stable reprogramming of bioenergetics (Fig. 1 P). Together, these data may suggest that bioenergetic regulation in MDA cells may depend on vinculin mechanosensing.

### Vinculin regulates migratory phenotypes and is required for directed cell migration in MDA-MB-231 cells

**Figure 2.**
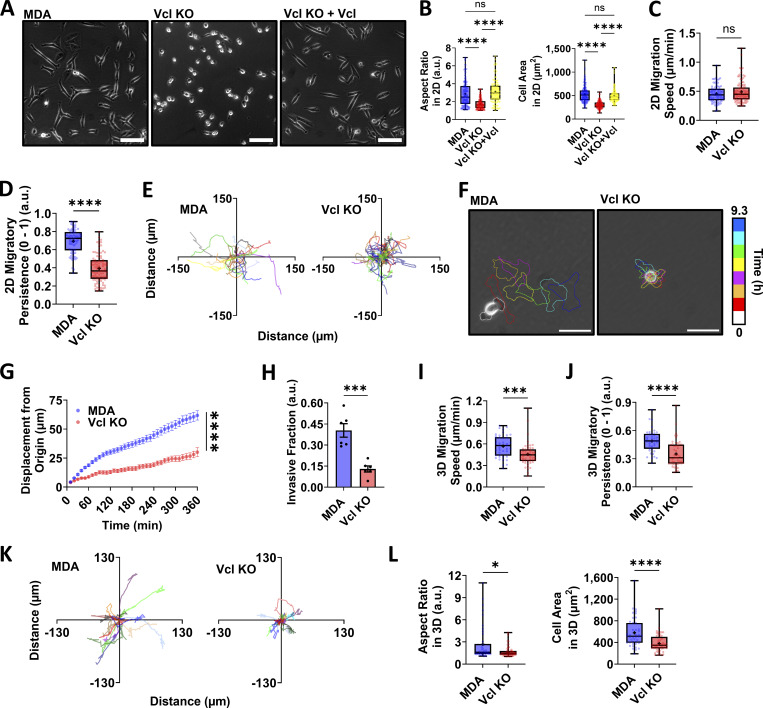
**Vcl KO does not increase migration speed, but reduces persistence of MDA-MB-231 cells. (A)** Representative phase-contrast images of cell lines indicated. Scale bar, 100 µm. **(B)** Quantification of cell aspect ratio and cell spread area on a 2D surface (*N* = 3, *n* > 108 cells). **(C and D)** Speed and (D) persistence of MDA and Vcl KO cells migrating on 2D surfaces. **(E)** Representative trajectories of cells moving on 2D surfaces. **(F)** Representative color-coded time lapses of cells migrating on 2D surfaces where each cell outline represents a 50-min interval from the previous outline. Scale bar, 50 µm. **(G)** Magnitude of displacement from origin at each time point. Note: some SEM bars are too small to display (*N* = 3, *n* = 78–95 cells for C–G). **(H)** Quantification of invasive fractions of MDA and Vcl KO cells through collagen-coated transwells (*N* = 3, *n* = 6). **(I and J)** Speed and (J) persistence of cells migrating in a 1.5 mg/ml collagen matrix (*N* = 3, *n* = 42–52 cells). **(K)** Representative XY trajectories of cells moving in a 1.5 mg/ml collagen matrix. **(L)** Cell aspect ratio and spread area in a 1.5 mg/ml collagen matrix (*N* = 3, *n* = 42–52 cells). The box-and-whisker plot shows median and 25th/75th percentile (box), min to max (whiskers), and mean (+). Bar graphs denote the mean ± SEM. XY plot shows the mean ± SEM. ns = not significant, *P < 0.05, ***P < 0.001, ****P < 0.0001.

Since metabolic activity increases in the absence of vinculin, we sought to determine how the cells utilize the energy produced by assaying cell migration, one of the most energy-demanding cellular processes. During cell migration, cells need energy to fuel reorganization of the actin cytoskeleton to support cell movement, and it is known that vinculin-deficient cells have increased actin flow rate, which may help to regulate migratory behavior, possibly by causing changes to cell protrusion behavior (Wu et al., 2021; Datta et al., 2021; Thievessen et al., 2013). In the absence of vinculin, cells appear rounder and less spread (Fig. 2, A and B), likely due to vinculin’s role in stabilizing the connection between integrins to the actin cytoskeleton (Ezzell et al., 1997). Differences in cell morphology are often associated with altered migration behavior, suggesting vinculin loss may shift migration patterns.

Interestingly, no significant differences in cell migration speed were detected in 2D in MDA cells with or without vinculin (Fig. 2 C), despite having observed bioenergetic and morphology changes. However, upon additional investigation of cell migration behavior, it was observed that the Vcl KO cells have reduced directional persistence during 2D cell migration (Fig. 2, D–F; and Videos 1 and 2). Although traveling at similar speeds to the parental MDA cells, Vcl KO cells traveled shorter distances over time (Fig. 2, E–G) due to their inability to persistently move in one direction. Together, our 2D migration data suggest that Vcl KO cells are not using increased bioenergetics to migrate more quickly; however, the increased bioenergetics could be supporting a cell phenotype that is promoting less persistent migration.

**Video 1.** **Representative phase-contrast time lapse of an MDA-MB-231 cell migrating in 2D, acquired on a Zeiss Axio Observer Z1 inverted microscope at 10-min intervals.** Time lapse depicts the MDA-MB-231 cell in Fig. 2 F. Scale bar, 50 µm. Playback, 7 fps.

**Video 2.** **Representative phase-contrast time lapse of an MDA-MB-231 Vcl KO cell migrating in 2D, acquired on a Zeiss Axio Observer Z1 inverted microscope at 10-min intervals.** Time lapse depicts the MDA-MB-231 Vcl KO cell in Fig. 2 F. Scale bar, 50 µm. Playback, 7 fps.

Since Vcl KO cells move less persistently than parental MDA cells in 2D and this correlated with increased energy production, we extended our migratory behavior investigation into 3D, where cells are not only in a more physiologically relevant environment, but also are more likely to move with greater persistence (Petrie et al., 2009). Carrying out our investigation in 3D enabled us to better understand whether the increased bioenergetics is being used to support a cell phenotype that is aiding in less persistent cell migration. When moving through an ECM-like 3D environment, cells often undergo morphological changes to deform and squeeze through confining regions of ECM. Such cell deformation involves cytoskeletal remodeling and thus requires cellular energy (Li et al., 2020; Yap and Kamm, 2005). Using a collagen-coated transwell with a serum gradient, we found that Vcl KO cells were less invasive through the collagen-coated transwell than the parental MDA cells (Fig. 2 H). These findings suggest that it is more difficult for MDA breast cancer cells lacking vinculin to travel through confining regions even despite their increased energetics. To further study 3D cell migration behavior, we embedded cells in 1.5 mg/ml collagen gels and measured migration rate. Unlike in 2D, Vcl KO cells move more slowly through a collagen gel (Fig. 2 I), likely because in 3D environments, the Vcl KO cells are not only faced with the absence of vinculin, and thus defects in cell–ECM adhesion, but they also need to navigate confining regions and complex architecture from the collagen. Collagen gels are softer than tissue culture plastic and primarily present collagen I ligands, so in addition to dimensionality differences, factors such as substrate stiffness and ECM composition may be contributing to the observed changes in relative migration speeds by affecting FA formation and therefore cell sensing of the matrix (Xia et al., 2024; Han et al., 2012). The reduced migration through the collagen-coated transwell and slower migration speed observed in metastatic breast cancer cells lacking vinculin in 3D agree with findings in mouse embryonic fibroblasts that the expression of full-length vinculin is essential for movement through fibronectin-coated transwells or invasion into 3D collagen matrices, as vinculin has been demonstrated to be essential for FA-mediated adhesion strength and the generation of forces required for cells to efficiently move through confining environments (Rothenberg et al., 2018; Mierke et al., 2010; Thievessen et al., 2015).

Consistent with 2D findings, the Vcl KO cells lack directional persistence during 3D migration (Fig. 2, J and K; and Videos 3 and 4) and have a rounder morphology relative to parental MDAs (Fig. 2 L). Together, these data indicate that while Vcl KO cells are more energetically active, their migration through collagen is not enhanced. Rather, the Vcl KO cells move with significantly less directional persistence than control cells and exhibit cell morphology changes, suggesting that vinculin may aid in promoting phenotypes that support more persistent migration.

**Video 3.** **Representative phase-contrast time lapse of an MDA-MB-231 cell moving in a 1.5 mg/ml collagen matrix, acquired on a Zeiss Axio Observer Z1 inverted microscope at 10-min intervals.** Time lapse supports data presented in Fig. 2, I–L. Scale bar, 20 µm. Playback, 7 fps.

**Video 4.** **Representative phase-contrast time lapse of an MDA-MB-231 Vcl KO cell moving in a 1.5 mg/ml collagen matrix, acquired on a Zeiss Axio Observer Z1 inverted microscope at 10-min intervals.** Time lapse supports data presented in Fig. 2, I–L. Scale bar, 20 µm. Playback, 7 fps.

### Vinculin regulates cell protrusion dynamics in a ROCK-dependent manner

**Figure 3.**
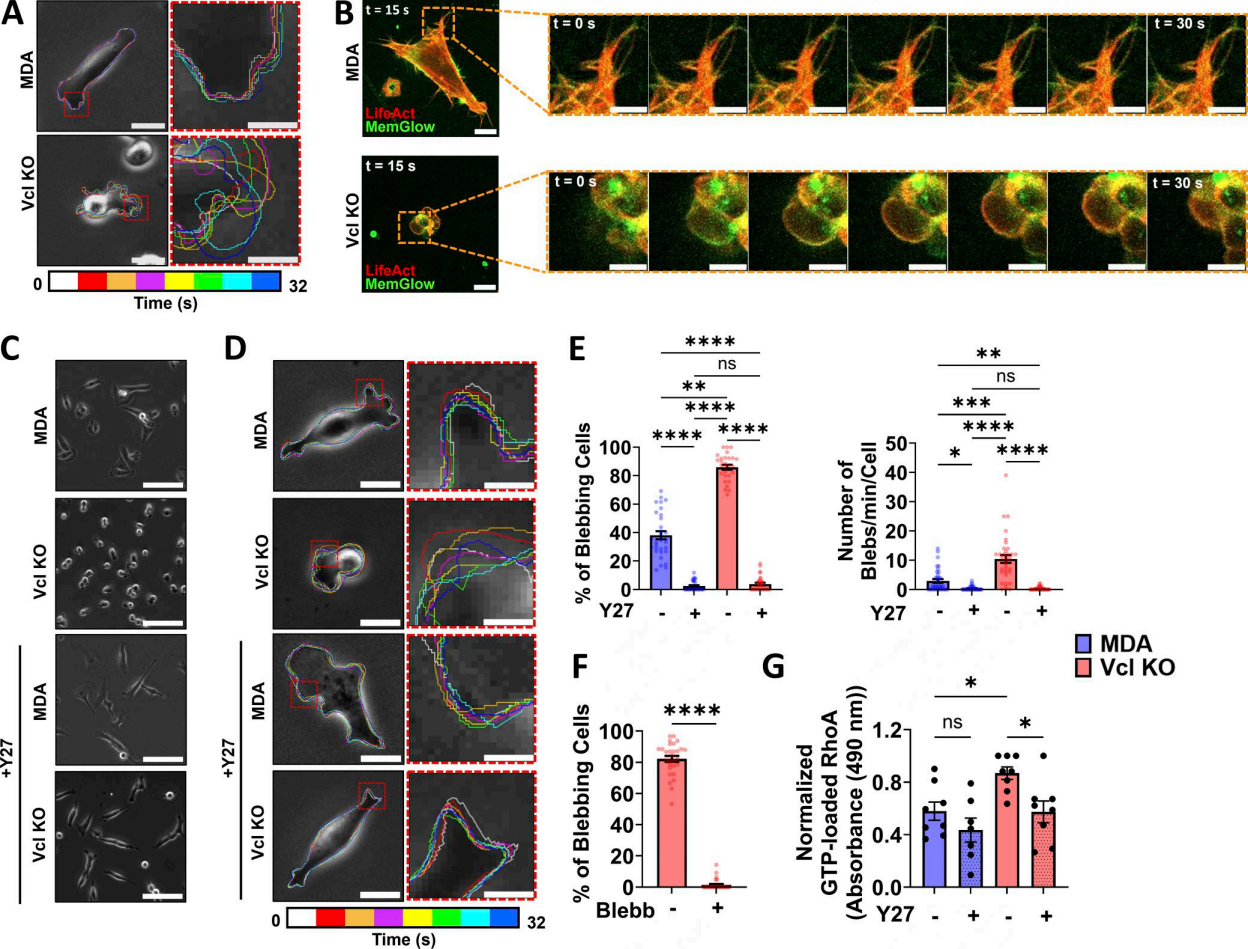
**Vcl KO increases GTP-RhoA–mediated ROCK activity and cell blebbing in MDA-MB-231 cells. (A)** Representative color-coded time lapse of MDA and Vcl KO cell shape on 2D surfaces, where each outline represents a 4-s interval. Scale bar, 20 µm in the image with the entire cell. Scale bar, 5 µm in the inset images in red boxes. **(B)** Representative time lapse indicating that blebs are forming in the Vcl KO cells as shown by separation of the membrane from the actin cortex labeled using MemGlow and LifeAct, respectively. Scale bar, 10 µm in the first image. Scale bar, 5 µm in the zoomed-in time-lapse images. **(C)** Representative phase-contrast images of cells treated with 20 µm Y-27632 (Y27) or the vehicle control (deionized water). Scale bar, 100 µm. **(D)** Representative color-coded time lapse of MDA and Vcl KO cell shape on 2D surfaces treated with 20 µm Y-27632 or the vehicle control. Scale bar, 20 µm in the image with the entire cell. Scale bar, 5 µm in the inset images in red boxes. For D, each outline represents a 4-s interval. **(E)** Quantification of the percentage of blebbing cells (*N* = 3, *n* = 28–31 fields of view) and the number of blebs per minute per cell (*N* = 3, *n* = 36 cells) for cells treated with 20 µm Y-27632 or the vehicle control. **(F)** Quantification of the percentage of blebbing cells in cells treated with 25 µm Blebb or the vehicle control (*N* = 3, *n* = 29 fields of view). **(G)** Normalized GTP-loaded RhoA measured using absorbance at 490 nm for cells treated with 20 µm Y-27632 or the vehicle control (*N* = 4). Bar graphs denote the mean ± SEM. ns = not significant, *P < 0.05, **P < 0.01, ***P < 0.001, ****P < 0.0001.

Since the Vcl KO cells did not use increased bioenergetics to support faster migration, but we observed rounder shapes and decreased migratory persistence during migration, we investigated the cell protrusion behavior as one potential explanation for the increased energy production in Vcl KO cells. Protrusions feed into determining both cell shape and persistence during migration (Datta et al., 2021; Wu et al., 2021; Vaidžiulytė et al., 2022). Given the role of vinculin in cell–ECM adhesion, our data may be suggesting that the Vcl KO cells are expending energy to support frequent protrusions and shape changes due to a reduced ability to adhere to the surface (Vaidžiulytė et al., 2022; Tsygankov et al., 2014). An increased rate of protrusions may require more remodeling of the actomyosin complex and thus could stimulate the cells to produce more energy to support such remodeling. Time-lapse microscopy revealed that the Vcl KO cells exhibit rounder and more dynamic protrusions in comparison with the parental MDA cells (Fig. 2 A and Fig. 3 A). The rounder-shaped protrusions on the Vcl KO cells were suggestive of bleb-like features. To confirm that the Vcl KO cells were sending out blebs, we imaged Vcl KO cells stably expressing LifeAct and stained with a membrane dye. The visible separation of the membrane from the actin cortex in the round-shaped protrusions, a commonly used approach to identify membrane blebs, suggested the presence of membrane blebs, which were not observed in the parental MDA cells (Fig. 3 B; and Videos 5 and 6) (Norman et al., 2010).

**Video 5.** **Representative time lapse of an MDA-MB-231 Vcl KO cell expressing LifeAct (red) and stained with 20 nM MemGlow (green), a membrane dye.** The time lapse was acquired on a Zeiss LSM800 confocal microscope at 5-s intervals and represents the MDA-MB-231 Vcl KO cell pictured in Fig. 3 B. Scale bar, 10 µm. Playback, 3 fps.

**Video 6.** **Representative time lapse of an MDA-MB-231 cell expressing LifeAct (red) and stained with 20 nM MemGlow (green), a membrane dye.** The time lapse was acquired on a Zeiss LSM800 confocal microscope at 5-s intervals and represents the MDA-MB-231 cell pictured in Fig. 3 B. Scale bar, 10 µm. Playback, 3 fps.

Cell blebbing is typically promoted by increased actomyosin contractility, often driven by active ROCK. We treated the cells with the ROCK inhibitor Y-27632 to determine whether ROCK activity may be promoting the dynamic blebbing in the Vcl KO cells (Kassianidou et al., 2017). Notably, Y-27632 treatment reduced the dynamic nature of the bleb-like protrusions and induced cell spreading in the Vcl KO cells (Fig. 3, C and D; and Videos 7, 8, 9, and 10). Quantification confirmed a decrease in the percentage of blebbing cells in the population and the number of blebs formed per minute by individual Vcl KO cells when ROCK was inhibited (Fig. 3 E). To further identify the mechanism through which ROCK may be influencing cell blebbing, we treated cells with Blebb to inhibit myosin II functionality, as myosin is downstream of ROCK. Like Y-27632 treatment, Blebb reduced the blebbing behavior observed in the Vcl KO cells (Fig. 3 F), suggesting that the blebbing activity in the absence of vinculin may occur in a ROCK/myosin II–dependent manner.

**Video 7.** **Representative phase-contrast time lapse of MDA-MB-231 cells moving in 2D, treated with vehicle control for Y-27632 (deionized water) for 20 h.** The time lapse was acquired on a Zeiss Axio Observer Z1 inverted microscope at ∼4-s intervals. A still image of this time lapse is provided in Fig. 3 C. Scale bar, 100 µm. Playback, 7 fps.

**Video 8.** **Representative phase-contrast time lapse of MDA-MB-231 Vcl KO cells moving in 2D, treated with vehicle control for Y-27632 (deionized water) for 20 h.** The time lapse was acquired on a Zeiss Axio Observer Z1 inverted microscope at ∼4-s intervals. A still image of this time lapse is provided in Fig. 3 C. Scale bar, 100 µm. Playback, 7 fps.

**Video 9.** **Representative phase-contrast time lapse of MDA-MB-231 cells moving in 2D, treated with 20 µM Y-27632 for 20 h.** The time lapse was acquired on a Zeiss Axio Observer Z1 inverted microscope at ∼4-s intervals. A still image of this time lapse is provided in Fig. 3 C. Scale bar, 100 µm. Playback, 7 fps.

**Video 10.** **Representative phase-contrast time lapse of MDA-MB-231 Vcl KO cells moving in 2D, treated with 20 µM Y-27632 for 20 h.** The time lapse was acquired on a Zeiss Axio Observer Z1 inverted microscope at ∼4-s intervals. A still image of this time lapse is provided in Fig. 3 C. Scale bar, 100 µm. Playback, 7 fps.

Since ROCK activity is promoted by active RhoA, or GTP-RhoA, we investigated whether Vcl KO cells have increased GTP-RhoA. Based on a G-LISA, Vcl KO cells have more active RhoA than parental MDA cells (Fig. 3 G), pointing to the potential role vinculin may play in mediating the activity of the RhoA/ROCK pathway. ROCK inhibition led to a reduction in GTP-RhoA in the Vcl KO cells, suggesting a feedback loop may be present since ROCK is a downstream effector of GTP-RhoA. Taken together, our data suggest that a more active RhoA/ROCK/myosin II pathway may be promoting the increased cell blebbing behavior observed in the absence of vinculin in breast cancer cells.

### Vinculin maintains reduced energy production in a ROCK/myosin II–dependent manner

**Figure 4.**
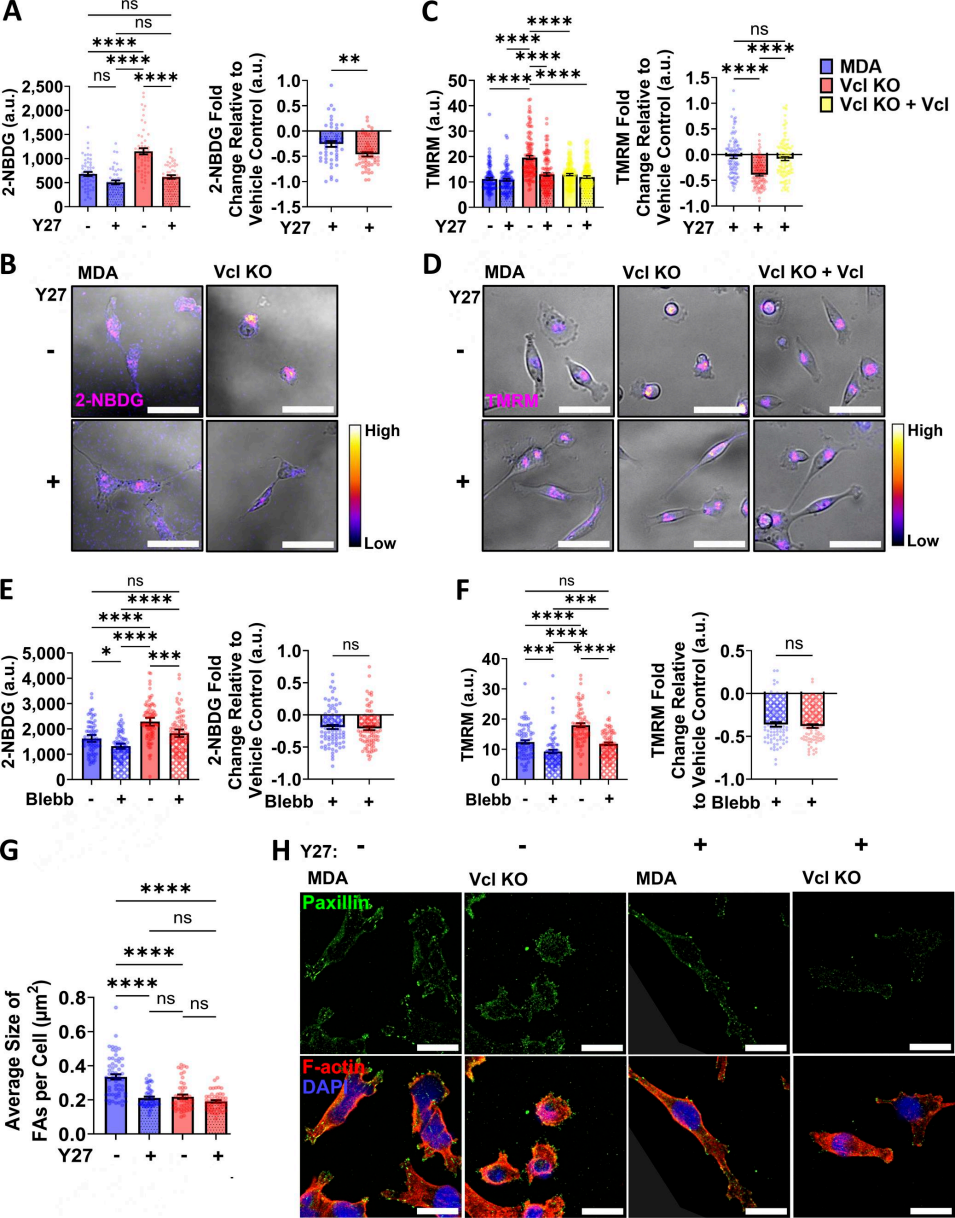
**Vinculin-mediated phenotypes are ROCK-dependent. (A)** Mean intensity of 2-NBDG for cells treated with 20 µM Y-27632 (Y27) or the vehicle control, and fold change of the 2-NBDG signal of cells treated with 20 µM Y-27632 relative to the vehicle control condition for each cell line (*N* = 3, *n* = 48–53 cells). **(B)** Representative images of 2-NBDG staining displayed using the Fire lookup table to indicate the relative intensity of the fluorescent signal. Scale bar, 50 µm. **(C)** Mean intensity of TMRM for cells treated with 20 µM Y-27632 or the vehicle control, and fold change of the TMRM signal of cells treated with 20 µM Y-27632 relative to the vehicle control condition for each cell line (*N* = 3, *n* = 95–118 cells). **(D)** Representative images of TMRM staining displayed using the Fire lookup table to indicate the relative intensity of the fluorescent signal. Scale bar, 50 µm. **(E)** Mean intensity of 2-NBDG for cells treated with 25 µM Blebb or the vehicle control, and fold change of the 2-NBDG signal of cells treated with 25 µM Blebb relative to the vehicle control condition for each cell line (*N* = 3, *n* = 80–88 cells). **(F)** Mean intensity of TMRM for cells treated with 25 µM Blebb or the vehicle control, and fold change of the TMRM signal of cells treated with 20 µM Blebb relative to the vehicle control condition for each cell line (*N* = 3, *n* = 76–84 cells). **(G)** Average area of FAs for cells treated with 20 µM Y-27632 or the vehicle control (*N* = 3, *n* = 46–50 cells). **(H)** Representative images of cells treated with 20 µM Y-27632 or the vehicle control and stained for paxillin (Pax), F-actin, and DAPI. Scale bar, 20 µm. Bar graphs denote the mean ± SEM. ns = not significant, *P < 0.05, **P < 0.01, ***P < 0.001, ****P < 0.0001.

Since our data indicate that Vcl KO cells have increased bioenergetics and exhibit fast, frequent blebbing behavior mediated by increased RhoA-ROCK activity, we sought to determine whether the increased RhoA-ROCK activity was promoting the greater energy production observed in the Vcl KO cells. When Vcl KO cells were treated with Y-27632 (Fig. 4, A–D) or Blebb (Fig. 4, E and F), glucose uptake and mitochondrial membrane potential decreased. Together, these data suggest that vinculin may aid in mediating cell bioenergetics via modulating the RhoA-ROCK-myosin II activity, likely due to the associated changes in protrusion dynamics and cell shape. In the absence of vinculin, cells may be expending energy to support an increase in actomyosin cytoskeletal reorganization associated with fast blebbing behavior and changes in cell shape. When a bleb-like protrusion expands, this is often attributed to rupturing of the membrane–cortex attachment, and then, the retraction process requires reassembly of the actin cortex beneath the plasma membrane (García-Arcos et al., 2024; Ikenouchi and Aoki, 2022). Considering that remodeling the actin cytoskeleton is an energetically demanding process, the frequent expansion and retraction of blebs are likely requiring increased energy production and utilization.

To investigate whether cell–ECM adhesion defects might be contributing to the protrusive phenotype in the KO cells in addition to increased ROCK activity, we assessed FA formation in the cells using paxillin staining, a widely used FA marker (Zhang et al., 2006; Thievessen et al., 2013). Vcl KO cells with and without Y-27632 treatment and parental MDAs with Y-27632 treatment all exhibited smaller FAs than parental MDA cells treated with the vehicle control (Fig. 4, G and H). These reductions in FA size suggest that defects in cell–ECM adhesion could be contributing to the phenotypes observed, although cell–ECM defects alone are not sufficient to promote the increased blebbing behavior and greater energy production observed in the Vcl KO cells. Together, our data indicate that the increased bioenergetics in Vcl KO cells may be fueling an increased rate of protrusion and retraction of membrane blebs, and these dynamic protrusions are promoted by greater activity of the RhoA/ROCK/myosin II pathway.

### Increased Rho activity and bioenergetics fuel cell protrusion dynamics

**Figure S3.**
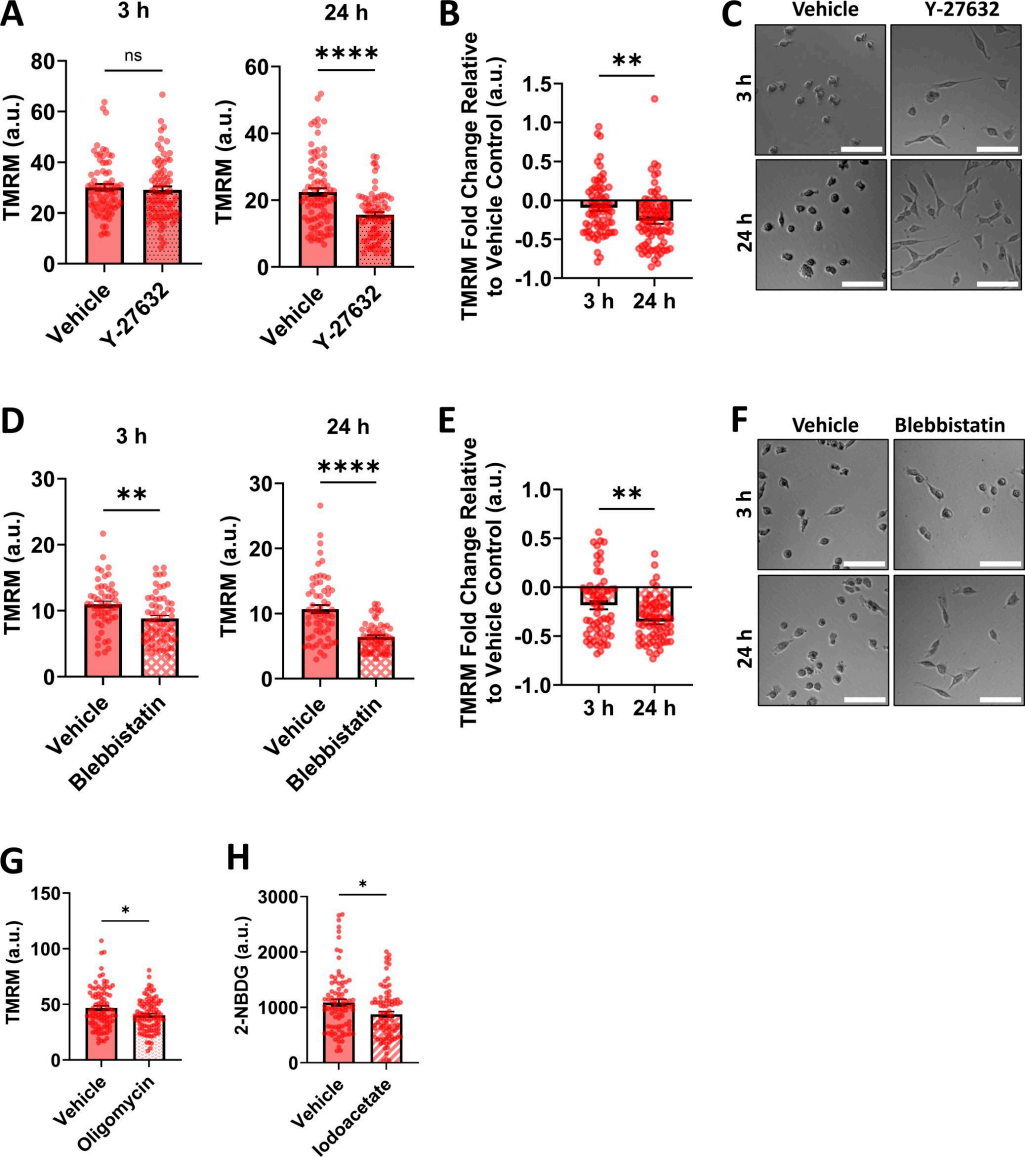
**Verifying cell responses to inhibitors. (A)** Verification of whether a short or long time point of 20 µm Y-27632 treatment results in bioenergetic changes in Vcl KO cells using TMRM as a metric for mitochondrial membrane potential. **(B)** Fold change of TMRM signal of the cells treated with 20 µm Y-27632 relative to the vehicle control (deionized water) condition at each time point (*N* = 3, *n* = 70–85 cells). **(C)** Representative images of Vcl KO cells treated with 20 µm Y-27632 or the vehicle control for short and long time points. Scale bar, 100 µm. **(D)** Verification of whether a short or long time point of 25 µm Blebb treatment results in bioenergetic changes in Vcl KO cells using TMRM as a metric for mitochondrial membrane potential. **(E)** Fold change of TMRM signal of the cells treated with 25 µm Blebb relative to the vehicle control (DMSO) condition at each time point (*N* = 3, *n* = 53–62 cells). **(F)** Representative images of Vcl KO cells treated with 25 µm Blebb or the vehicle control for short and long time points. Scale bar, 100 µm. **(G and H)** Verification that cell treatment with (G) 20 µm oligomycin (*N* = 3, *n* = 90–96 cells) or (H) 10 µm iodoacetate (*N* = 3, *n* = 86 cells) treatments induced decreases in cell energy metrics. Bar graphs denote the mean ± SEM. ns = not significant, *P <0.05, **P < 0.01, ****P < 0.0001.

**Figure 5.**
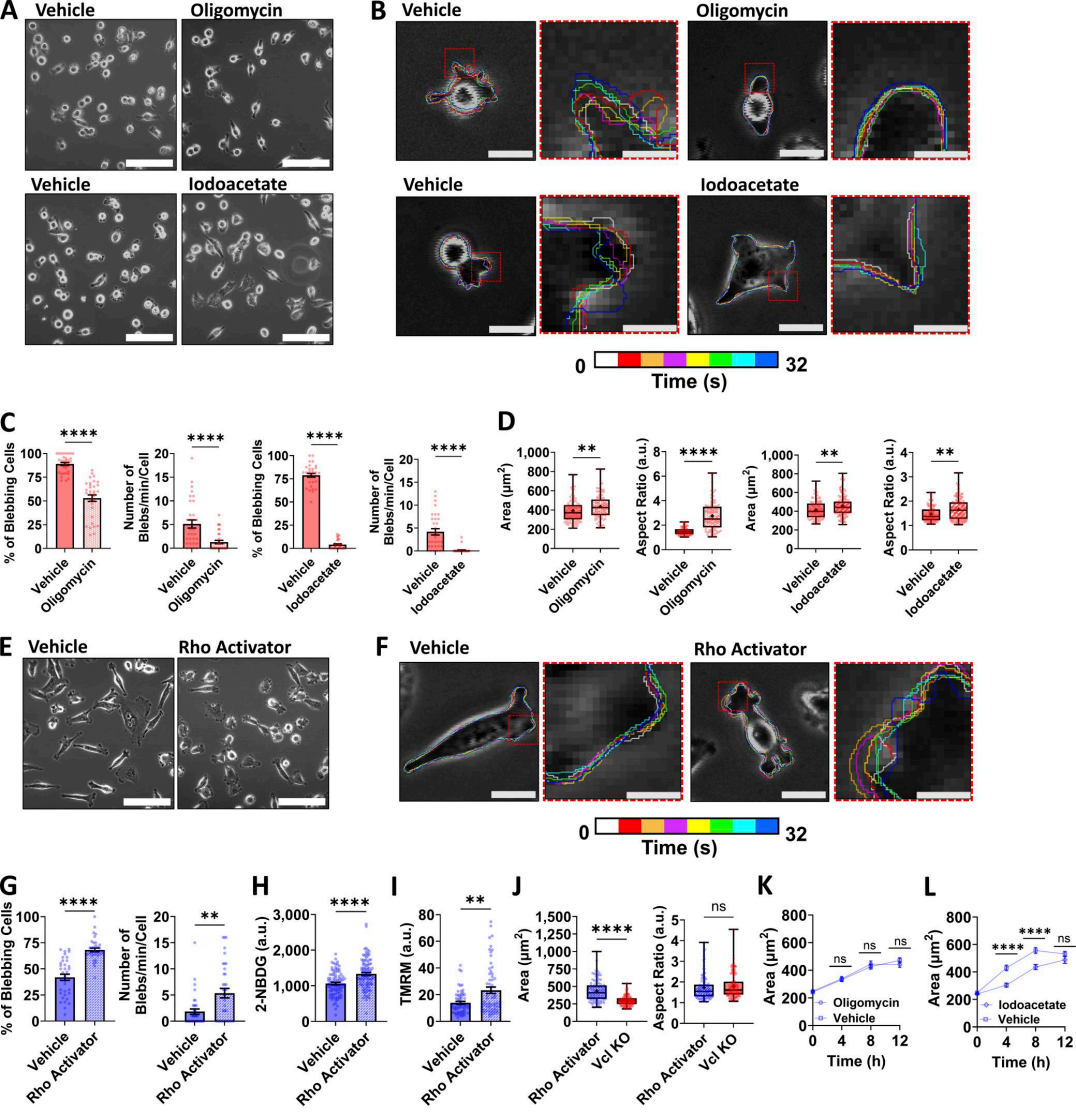
**Metabolic inhibition of Vcl KO cells reduces cell blebbing, and Rho activation of parental MDA-MB-231 cells increases cell blebbing and bioenergetics. (A and B)** Representative (A) phase-contrast images (scale bar, 100 µm) and (B) color-coded time lapses of Vcl KO cells treated with oligomycin, iodoacetate, or the vehicle control for each drug. Scale bar, 20 µm in the image with the entire cell. Scale bar, 5 µm in the inset images in red boxes. **(C)** Percentage of blebbing cells treated with oligomycin (*N* = 3, *n* = 33–34 fields of view), iodoacetate (*N* = 3, *n* = 31 fields of view), or the vehicle and the number of blebs per cell per minute for Vcl KO cells treated with oligomycin (*N* = 3, *n* = 32–33 cells), iodoacetate (*N* = 3, *n* = 32–34 cells), or the vehicle control. **(D)** Cell spread area of Vcl KO cells treated with oligomycin (*N* = 3, *n* = 92–97 cells), iodoacetate (*N* = 3, *n* = 101–137 cells), or the vehicle control, and the aspect ratio of Vcl KO cells treated with oligomycin (*N* = 3, *n* = 90–91 cells), iodoacetate (*N* = 3, *n* = 94–134 cells), or the vehicle control. **(E and F)** Representative (E) phase-contrast images (scale bar, 100 µm) and (F) color-coded time lapses of parental MDA cells treated with Rho activator or the vehicle control. Scale bar, 20 µm in the image with the entire cell. Scale bar, 5 µm in the inset images in red boxes. **(G)** Percentage of blebbing cells (*N* = 3, *n* = 36 fields of view) or number of blebs per cell per minute (*N* = 3, *n* = 36 cells) for MDA cells treated with Rho activator or the vehicle control. **(H)** Mean intensity of 2-NBDG in parental MDA cells treated with Rho activator or the vehicle control (*N* = 3, *n* = 90–97 cells). **(I)** Mean intensity of TMRM in parental MDA cells treated with Rho activator or the vehicle control (*N* = 3, *n* = 61–62 cells). **(J)** Cell spread area (*N* = 3, *n* = 106–140 cells) and aspect ratio (*N* = 3, *n* = 106–140 cells) of parental MDA cells treated with the Rho activator compared with Vcl KO cells. **(K)** Cell spread area of parental MDA cells being treated with 20 µm oligomycin or the vehicle control over time after trypsinization (*N* = 3, *n* = 74–89 cells). **(L)** Cell spread area of parental MDA cells being treated with 10 µm iodoacetate or the vehicle control over time after trypsinization (*N* = 3, *n* = 72–87 cells). For K and L, time of 0 h indicates when cells are initially seeded on tissue culture surface and not yet adhered or spread. Bar graphs denote the mean ± SEM. The box-and-whisker plot shows median and 25th/75th percentile (box), min to max (whiskers), and mean (+). XY plots show the mean ± SEM. ns = not significant, **P < 0.01, ****P < 0.0001.

Since our data suggest that without vinculin ROCK activity increases to promote cell blebbing and increased bioenergetics, we sought to determine whether cell protrusion dynamics and bioenergetics are interdependent. To investigate the relationship between blebbing protrusions and bioenergetics, we treated the Vcl KO cells with metabolic inhibitors to reduce bioenergetics (Fig. S3, G and H) and assessed cell blebbing. To inhibit metabolism, we utilized oligomycin and iodoacetate, which inhibit ATP synthase in the electron transport chain and GAPDH in glycolysis, respectively. Both inhibitors reduced the frequency of cell blebbing behavior in the Vcl KO cells with a greater decrease in blebbing observed using iodoacetate treatment compared with oligomycin treatment (Fig. 5, A–C). The reductions in cell blebbing allowed increased cell spread area and elongation (Fig. 5 D). These findings suggest that increased energy production may fuel cell blebbing and that glycolysis may play a bigger role in fueling cell blebbing than the electron transport chain.

Since the Vcl KO cells have increased RhoA-ROCK activity, bioenergetics, and blebbing relative to the parental MDAs, we sought to understand whether pharmacologically stimulating RhoA-ROCK activity in parental MDA cells will lead to greater energy production and cell blebbing. We treated parental MDA cells with a Rho activator since ROCK is an effector for active RhoA, which promoted blebbing (Fig. 5, E–G) and increased glucose uptake and mitochondrial membrane potential, likely to help fuel the increased blebbing behavior (Fig. 5, H and I). Although treatment with a Rho activator decreased the aspect ratio in MDA cells to become similar to that of the Vcl KO cells, the MDA cells treated with the Rho activator maintained an increased spread area relative to the vinculin-deficient population, despite the initiation of blebbing behavior, likely due to the important role of vinculin in promoting cell–ECM adhesion (Fig. 5 J). Taken together, these data support that increased RhoA-ROCK activity can induce bleb formation and fuel greater cell protrusion dynamics and that it may be linked to increased cell bioenergetics even in the presence of vinculin, similar to the effects observed with vinculin deficiency in MDA cells.

Since our data suggest that cell blebbing, spreading, and bioenergetics may be linked, we investigated the effect of metabolic inhibitors on the spreading rate of parental MDA cells following trypsinization. While oligomycin did not affect the spreading rate of MDA cells, iodoacetate treatment caused MDA cells to spread more slowly (Fig. 5, K and L). Given the targets of oligomycin and iodoacetate, our data may suggest that glycolysis is more important for cell spreading than the electron transport chain.

Here, we established a relationship between vinculin, cell bioenergetics, and migration behavior, and the RhoA/ROCK/myosin II pathway in MDA cells. Our findings that the greater energy production in Vcl KO cells did not confer a migratory advantage are notable since migration processes typically require coordinated activity of the cytoskeleton and substantial amounts of energy, and vinculin is pivotal for regulating dynamics of the actin cytoskeleton (Jannie et al., 2015; Thievessen et al., 2013). Instead, the differences in cell migration behavior and morphology led us to find that vinculin loss led to dynamic blebbing and increased energy production, both of which were suppressed by ROCK and myosin II inhibition. Since actin remodeling is energy-intensive, our findings suggest that reassembly of the actin cortex associated with frequent expansion and retraction of blebs is likely the cause of increased energy production and utilization in cells with high RhoA-ROCK activity (Ikenouchi and Aoki, 2022; García-Arcos et al., 2024).

The activity of Rho GTPases, like RhoA, is regulated by guanine nucleotide exchange factors (GEFs) and GTPase-activating proteins (GAPs), which can be recruited to FAs. Although FA proteins such as talin, FAK, and paxillin can directly interact with GEFs or GAPs, a direct interaction with vinculin has not been established (Tomar and Schlaepfer, 2009; Haining et al., 2018; López-Colomé et al., 2017). However, the increased energy production observed in the Vcl KO cells persisted until long-term treatment with Y-27632 or Blebb (Fig. S3, A–F). The requirement of long-term treatments may suggest that alterations in gene expression could underlie the bioenergetic shift, such as genetic changes associated with altered expression of adhesion-based or blebbing-based migration machinery. Thus, future work should further investigate the mechanisms through which the bioenergetic shifts occurred, as it may be consistent with changes in gene expression and not fast GEF/GAP-mediated changes in RhoA activity.

The role of FA proteins in regulating cell metabolism has become an area of emerging interest (Fabiano et al., 2025). For example, knockdown in MDA cells of FA protein talin 2, which can directly bind F-actin, reduced basal levels of glucose and lactic acid and inhibited cell invasion and migration (Wen et al., 2019). In our study, Vcl KO increased glucose uptake, which may suggest that specific FA proteins could differentially impact cell metabolism, potentially due to their distinct roles at FAs. Talin promotes FA formation by activating integrins, while vinculin is thought to be more important for FA stability (Kechagia et al., 2019; Humphries et al., 2007; Peng et al., 2011). Recent studies also link FA proteins to metabolic machinery, including glucose transporters and mitochondria with FA proteins. Talin 1 has been linked to GLUT-4 expression in patients with polycystic ovary syndrome, and a co-immunoprecipitation assay showed that talin 1 interacts with GLUT-4 (Li et al., 2023). Further, the expression of vinculin mutations in MCF-7 cells that enhance (Vcl-T12, constitutively active vinculin) or inhibit mechanosignaling (Vcl-T, binds to the barbed ends of F-actin to prevent actin polymerization) causes increased perinuclear mitochondrial clustering or increased uniform distribution of mitochondria, respectively, depending on substrate stiffness (Daga et al., 2024). Unlike talin 1, vinculin has not been shown to directly regulate the expression of glucose transporters. Future work is needed to elucidate the mechanisms through which FA proteins regulate metabolic machinery, but perhaps the distinct molecular processes that different FA proteins are involved in at FAs have altered energetic requirements.

In summary, our work suggests a previously unidentified mechanism by which vinculin influences the bioenergetic phenotype of cells via the RhoA/ROCK/myosin II pathway. Our study deepens our understanding of the molecular mechanisms by which deregulated bioenergetics dictate cell behavior, an intersection crucial for understanding cancer progression. Notably, several ROCK inhibitors are approved for clinical use, including in the United States, and there have been over a dozen recent clinical trials using ROCK inhibitors, such as Y-27632, since ROCK inhibitors used in preclinical models have demonstrated promising results (Barcelo et al., 2023). The potential for clinical translation is also an emerging area in the context of immune cells, as recent work has begun to uncover how changes to cell–ECM sensing, through changes to substrate stiffness, can reprogram the metabolism of tumor-associated macrophages to impair CD8^+^ T cell function (Tharp et al., 2024), and increased ECM stiffness may also enhance T cell activation and oxidative phosphorylation rates (Shi et al., 2023). Given the potential for clinical impact, future studies should continue to investigate the mechanistic links between cell–ECM adhesion, metabolism, and cell behavior, as a deeper understanding may help in identifying effective druggable targets for the treatment of a range of disease states.

## Materials and methods

### Cell culture and reagents

Highly metastatic MDA-MB-231 breast adenocarcinoma cells (HTB-26; ATCC), HEK-293T cells (CRL-3216; ATCC), and NIH/3T3 fibroblasts (CRL-1658) were maintained at 37°C and 5% CO_2_ in Dulbecco’s modified Eagle’s media (DMEM; 11965092; Thermo Fisher Scientific) supplemented with 10% fetal bovine serum (FBS; Atlanta Biologicals) and 1% penicillin–streptomycin (Thermo Fisher Scientific). MCF10A mammary epithelial cells (CRL-10317) were maintained in DMEM supplemented with 5% horse serum, 20 ng/ml hEGF (all from Invitrogen), 0.5 µg/ml hydrocortisone, 10 µg/ml insulin, 100 ng/ml cholera toxin (all from Sigma-Aldrich), and 1% penicillin–streptomycin. To inhibit ROCK, cells were treated with a commonly used dose of 20 µM Y-27632 (688000; Sigma-Aldrich) for 20 h (Kawada et al., 1999; Kurosawa, 2012; Sivasubramaiyan et al., 2009). To inhibit myosin II, cells were treated with a commonly used dose of 25 µM Blebb (Selleck Chemicals) for 20 h (Lin et al., 2018; Aermes et al., 2021). 20-h treatment times for Y-27632 and Blebb were used, as a long time point resulted in both cell morphology and bioenergetic changes (Fig. S3, A–F).

### RNA interference

MDA-MB-231 cells stably co-expressing PercevalHR (#49083; Addgene) and pHRed (#31473; Addgene) probes, gifts from Gary Yellen (Harvard Medical School, Boston, MA, USA), were transfected with 50 pmol/µl nontargeting scrambled control siRNA (AM4611; Thermo Fisher Scientific) or siRNA targeting Vcl (AM51331 [siRNA ID: 108455] and AM16708 [siRNA ID: 108453]; Thermo Fisher Scientific) using Lipofectamine 2000 (11668030; Thermo Fisher Scientific) and Opti-MEM transfection medium (31985062; Thermo Fisher Scientific) according to the manufacturer’s instructions. For PercevalHR measurements, cells were imaged 48 h after transfection by collecting z-stacks on a Zeiss LSM800 confocal microscope using a 20×/0.8 NA objective in an environmental chamber maintained at 37°C and 5% CO_2._ For TMRM and 2-NBDG measurements, parental MDA-MB-231 cells were transfected with siRNA and then treated with media containing 20 nm TMRM or 0.146 mM 2-NBDG to measure mitochondrial membrane potential or glucose uptake at 48 h after transfection.

### Generation of Vcl KO cell line

Generation of the MDA-MB-231 Vcl KO cell line was completed as described previously (Mosier et al., 2023). Briefly, MDA-MB-231 cells were transfected with CRISPR/Cas9 ribonucleoprotein complexes consisting of *Streptococcus pyogenes* Cas9 predesigned multiguide RNAs targeting Vcl (Gene KO Kit v2; Synthego) using electroporation (Bio-Rad Gene Pulser). Single-cell clones were assayed using western blotting and Sanger sequencing (Azenta Life Sciences) to validate Vcl KO.

For generation of the NIH/3T3 and MCF10A Vcl KO cell lines, cells were transfected using electroporation as described for the MDA-MB-231 cells, except clones were not generated. For the NIH/3T3 cells, the electroporation settings used for transfection were 120 V and 950 µF. For the MCF10A cells, the electroporation settings used for transfection were 100 V and 1,000 µF. Transfected populations were assayed for knockout using western blotting to verify reduction in vinculin protein expression.

### Generation of Vcl rescue cell line

To produce lentivirus, HEK-293T cells were transfected with lentiviral pRRL-Vcl-mVenus or empty vector pRRL together with second-generation packaging constructs psSPAX2 and pMD2.G in TransIT-LT1 (MIR 2304; Mirus). Lentiviral particles were harvested 48 and 72 h after transfection and concentrated with Lenti-X Concentrator (631232; Takara Bio). Vcl KO cells were transduced with pRRL-Vinculin-mVenus or pRRL lentiviral particles in the presence of 8 µg/ml polybrene (sc-134220; Santa Cruz Biotechnology) to generate the Vcl rescue and empty vector control cell lines, respectively. mVenus+ cells were collected using fluorescence-activated cell sorting at the Vanderbilt Flow Cytometry Shared Resource, and verification of physiologically relevant levels of Vcl expression was done using western blotting, in addition to confirming that majority of the cells sorted were expressing the mVenus-containing plasmid using the Zeiss LSM800 confocal microscope.

### Western blotting

Cells were rinsed with phosphate-buffered saline (PBS) and lysed using preheated (90°C) 2× sodium dodecyl sulfate (SDS) lysis buffer. Lysates were resolved by SDS–polyacrylamide gel electrophoresis and transferred to polyvinylidene fluoride membranes. For verification of siRNA knockdown, Vcl KO, and Vcl re-expression in the Vcl KO cells, transferred membranes were blocked with 5% milk in TBS/Tween for 1 h and then incubated overnight with a mouse anti-vinculin antibody (05-386; Millipore) at 1:1,000 or a mouse anti-β-actin antibody (A1978; Sigma-Aldrich) at 1:1,500 in 5% milk in TBS/Tween at 4°C. After three washes, membranes were then incubated with either 800 IRDye goat anti-mouse secondary antibody (431386; LI-COR) at 1:5,000 or horseradish peroxidase–conjugated secondary antibody at 1:2,000 in 5% milk in TBS/Tween for 1 h at room temperature and then washed three times. IRDye-stained membranes were then imaged with an Odyssey Fc (LI-COR Biosciences). Horseradish peroxidase–conjugated secondary antibody–stained membranes were incubated with SuperSignal West Pico Chemiluminescent Substrate (Thermo Fisher Scientific) and then imaged immediately using a ChemiDoc imaging system (Bio-Rad).

### Quantification of intracellular ATP:ADP ratio

MDA-MB-231 cells expressing PercevalHR and pHRed and treated with siRNA targeting Vcl or the scrambled control were imaged in media using Zeiss LSM800 confocal microscope operated by Zen software and equipped with a 20×/0.8 NA objective and an environmental chamber maintained at 37°C and 5% CO_2_. The quantification of the intracellular ATP:ADP ratio was calculated as described previously (Tantama et al., 2013; Zanotelli et al., 2018). Briefly, background pixel intensity was measured for each channel from each image and subtracted to minimize obstruction from background noise using Fiji/ImageJ software (NIH, 2.14.0/1.54f) (Schindelin et al., 2012). A region of interest was drawn around each cell, and the mean pixel intensity of the cell was acquired to calculate ATP, ADP, pH1, and pH2 intensities. The PercevalHR ratios were found by dividing the ATP by the ADP intensity for each cell. The pH1 intensity was divided by the pH2 intensity to calculate the pHRed ratio for that cell. The PercevalHR sensor is pH-sensitive, so a pH calibration was done to remove pH bias, as described previously (Tantama et al., 2011). Briefly, intracellular pH was varied by adding NH_4_Cl to the cell media to introduce alkalization, and the ATP:ADP ratio was assumed to be maintained constant during the short calibration period. A linear correlation is then made between the uncorrected PercevalHR signal and pHRed signal to predict pH bias. The linear relationship is used to transform pHRed signal to predict changes in PercevalHR due to a solely change in pH. The uncorrected PercevalHR value was divided by the transformed pH-corrected signal to approximately correct for pH artifacts for normalization.

### 3D cell embedding in collagen

For 3D collagen matrix studies, 3D collagen matrices were prepared using type I collagen extracted from rat tail tendons as described previously (Rahman et al., 2016; Zanotelli et al., 2018). Briefly, 50,000 cells were embedded in 500 µl of a 1.5 mg/ml collagen solution in a 24-well plate. The 1.5 mg/ml collagen solution was prepared from a 10 mg/ml collagen stock solution diluted with ice-cold complete media and neutralized to pH 7.0 with 1N NaOH. Gels were allowed 45 min to polymerize, and then, an additional 500 μl of media was added to feed the cells and to hydrate the collagen gel. For all experiments, cells were allowed to adhere and spread overnight in the collagen matrix maintained at 37°C and 5% CO_2_ before imaging on the Zeiss LSM800 confocal microscope operated by Zen software. For all imaging done of cells in 3D collagen gels, cells were imaged in the range of 200–250 µM from the bottom surface to avoid effects on the cells from the bottom of the plate.

### Quantification of glucose uptake

To measure glucose uptake in 2D, cells were cultured for 9.5 h on glass coverslips and then incubated with phenol red–free complete media supplemented with 0.146 mM 2-NBDG and incubated at 37°C and 5% CO_2_ for 30 min. Then, cells were fixed with 3.2% paraformaldehyde for 10 min at room temperature. To reduce background, cells were washed overnight in 1× PBS at 4°C. After washing, coverslips were then mounted using mounting solution (H100010; Thermo Fisher Scientific) and imaged by collecting z-stacks using a 20×/0.8 NA objective on a Zeiss LSM800 confocal microscope operated by Zen software.

For Y-27632 and Blebb experiments, cells were cultured overnight on glass coverslips and then treated with 20 µm Y-27632, 25 µM Blebb, or the vehicle control for 19.5 h. Media were replaced with phenol red–free complete media supplemented with 0.146 mM 2-NBDG and 20 µm Y-27632, 25 µM Blebb, or the vehicle control and incubated for 30 min. Then, cells were fixed with 3.2% paraformaldehyde for 10 min at room temperature and washed overnight in 1× PBS at 4°C to reduce background, and then, coverslips were mounted using mounting solution (H100010; Thermo Fisher Scientific). Samples were imaged by collecting z-stacks using a 20×/0.8 NA objective on a Zeiss LSM800 confocal microscope operated by Zen software.

Measuring glucose uptake in 3D collagen was done as described previously (Zanotelli et al., 2018), with some modifications. After embedding cells in a 1.5 mg/ml collagen matrix using phenol red–free complete media and allowing cells to adhere overnight, cells were incubated in 0.146 mM 2-NBDG in phenol red–free complete media for 24 h by replacing the media on top of the collagen gel. Then, cells were fixed with 3.2% paraformaldehyde for 10 min at room temperature followed by an overnight wash with 1× PBS at 4°C to reduce background. Cells were then imaged by collecting z-stacks of individual cells using a 40×/1.1 NA water immersion objective on a Zeiss LSM800 confocal microscope operated by Zen software.

For the adherent and floating cell experiments, cells were grown on either a tissue culture–treated surface or ultra-low attachment plates (3473; Corning), and were then incubated with 0.146 mM 2-NBDG for 30 min and lifted off of the plates and fixed immediately. After washing the stained cell pellet with PBS, the fixed cells were spun onto glass slides using a CytoSpin 3 (Shandon), mounted, then imaged by collecting z-stacks using a 20×/0.8 NA objective on a Zeiss LSM800 confocal microscope operated by Zen software. Cells were processed with the cytospin approach since the cells on the ultra-low attachment plates were floating in the wells and could not be accurately imaged, and we wanted to treat all the samples in the same way to have comparable measurements.

To calculate 2-NBDG uptake in 2D and 3D, z-stacks were projected, and then, cells were manually outlined, and mean pixel intensity was calculated after background subtraction using Fiji software.

### Quantification of mitochondrial membrane potential

To quantify mitochondrial membrane potential in 2D, cells were cultured for 9.5 h on glass-bottom 12-well plates before active mitochondria were stained by incubating cells in complete media supplemented with 20 nM TMRM for 30 min at 37°C and 5% CO_2_. Cells were then imaged by collecting z-stacks using a Zeiss LSM800 confocal microscope operated by Zen software and equipped with a 20×/0.8 NA objective and an environmental chamber maintained at 37°C and 5% CO_2_.

For Y-27632 and Blebb experiments, cells were allowed to adhere overnight on glass-bottom 12-well plates and then treated with 20 µm Y-27632, 25 µM Blebb, or the vehicle control for 19.5 h. Media were replaced with complete media supplemented with 20 nM TMRM and 20 µm Y-27632, 25 µM Blebb, or the vehicle control and incubated for 30 min. Cells were then imaged by collecting z-stacks using a Zeiss LSM800 confocal microscope operated by Zen software and equipped with a 20×/0.8 NA objective and an environmental chamber maintained at 37°C and 5% CO_2_.

For TMRM measurements in 3D, cells were embedded in a 1.5 mg/ml collagen matrix as described above, with modification. Here, the cells were seeded in the collagen matrix and fed with media containing 75 nM TMRM after the 45-min collagen polymerization step. After 19–20 h of incubation at 37°C and 5% CO_2_, the cells were imaged by collecting z-stacks using a Zeiss LSM800 confocal microscope operated by Zen software and equipped with a 40×/1.1 NA water immersion objective and an environmental chamber maintained at 37°C and 5% CO_2_.

To calculate mitochondrial membrane potential in 2D and 3D, z-stacks were projected, cells were manually outlined, and then mean pixel intensity was calculated after background subtraction using Fiji software, as described previously (Wu et al., 2021).

### Aspect ratio and cell area measurements

After cells were seeded on cell culture–treated plastic and allowed to adhere and spread overnight, phase-contrast images of the cells were collected using a Zeiss Axio Observer Z1 inverted microscope operated by AxioVision software and equipped with a Hamamatsu ORCA-ER, a 10×/0.3 NA objective, and an environmental chamber maintained at 37°C and 5% CO_2_. Analysis of aspect ratio and cell area was done by manually outlining cells using Fiji software.

### Cell viability

To assess cell viability, cells were seeded at a density of 15,000 cells/well in a 48-well plastic well plate. Cells were incubated overnight, and then, viability was studied using a LIVE/DEAD kit (R37601; Thermo Fisher Scientific) according to the manufacturer’s instructions. 10 random fields of view were captured for each biological replicate using a Zeiss LSM800 confocal microscope operated by Zen software and equipped with a 10×/0.3 NA objective and an environmental chamber maintained at 37°C and 5% CO_2_.

### EdU proliferation assay

Cells were cultured overnight before receiving treatment with EdU dye solution (C10337; Thermo Fisher Scientific) and fixed using 3.2% PFA. The assay was performed following the manufacturer’s protocol, in addition to staining the nuclei with DAPI. At least 10 random fields of view were captured for each biological replicate using a 10×/0.3 NA objective on a Zeiss LSM800 confocal microscope operated by Zen software. The percentage of EdU incorporation was quantified using Fiji software as the percentage of cells positive for EdU relative to the total cell count using DAPI.

### 2D and 3D cell migration seeding

For 2D migration studies, cells were seeded on cell culture–treated plastic in complete media and allowed to adhere and spread for 6 h before starting time-lapse imaging. Time lapses were collected using an interval of 10 min between frames, and analysis was done by measuring the cells 6–12 h after seeding. For 3D migration studies, 3D collagen matrices were prepared as described above. Quantification was completed using 6 h of time-lapse imaging with 10-min intervals between frames, such that cells were measured 15–21 h after seeding to allow cells time to settle and spread in the collagen. To remove outliers for the total distance traveled from the origin and from the rose plots, the Identify Outliers test was used in Prism on the MDA and Vcl KO cells for the dataset that sums up the distance to report the total distance traveled. Both the 2D and 3D cell migration time-lapse cells were imaged using the 10×/0.3 NA objective on a Zeiss Axio Observer Z1 inverted microscope operated by AxioVision software and equipped with a Hamamatsu ORCA-ER camera and an environmental chamber maintained at 37°C and 5% CO_2_.

### 2D and 3D cell migration analysis

Cell migration speed was measured by manually outlining cells using Fiji software and calculating the displacement of the cell centroid (from frame to frame in the time-lapse series) dividing by the time interval as described previously (Mosier et al., 2019, 2023). Persistence was quantified by first calculating a persistence value for each 1-h interval. This was done by calculating the displacement from the first to last point over 1 h divided by the total distance traveled for that 1 h (sum of centroid displacements between 10-min time steps). This was repeated 6 times for each cell to account for a total of 6 h of migration. We then averaged the persistence value for each 1-h time step to report an average persistence value between 0 and 1 for each cell, where 1 is most persistent, a metric used previously (Mosier et al., 2023). Cells that divided or interacted with other cells during migration were excluded from migration analysis. Data from individual cells were pooled from at least three independent experiments, and data for all single cells are represented on the plots, as done previously (Mosier et al., 2019; Mosier et al., 2023; Rahman et al., 2016).

### Collagen transwell assay

Cells were seeded on top of a transwell insert (Greiner Bio-One) with 8-µm pores coated with 1 mg/ml collagen. A serum gradient was established by adding DMEM supplemented with 0.5% FBS on the top of transwell inserts and complete media (10% FBS) at the bottom. Cells were allowed to migrate through the transwell insert for 4 days. Cells were then collected from the top and bottom chambers of the transwell, and the invasive fraction of the cells was calculated as the fraction of cells that migrated through the collagen-coated transwell divided by the total number of cells, as described in previous studies (Hapach et al., 2021; Schwager et al., 2022).

### ROCK inhibitor time lapses

For ROCK inhibitor experiments, cells in complete media were treated with 20 µM Y-27632 or the vehicle control (deionized water) for 20 h. Treatment time was determined based on when a difference in cell morphology and energetics was observed (Fig. S3). Then, time-lapse videos were also collected using the 10×/0.3 NA objective on a Zeiss Axio Observer Z1 inverted microscope operated by AxioVision software and equipped with a Hamamatsu ORCA-ER camera and an environmental chamber maintained at 37°C and 5% CO_2_. Time lapses were collected over a 5-min duration with ∼4 s between frames. Color-coded time lapses were generated by manually outlining cells using Fiji software.

### Cell blebbing quantification

For cell blebbing quantification, time-lapse videos of the cells were used. To assess the percentage of blebbing cells, quantification was performed on 10–12 fields of view per condition over a 5-min period per replicate. To quantify the number of blebs per minute per cell, cells were randomly selected from various fields of view and analyzed over a 1-min period by manually counting the number of blebs expanded and retracted.

### Measuring GTP-RhoA

GTP-RhoA was assessed using a G-LISA kit following the manufacturer’s protocol (BK124; Cytoskeleton). Absorbance was measured at 490 nm using the Cytation 5 (Agilent), and values were normalized for each replicate.

### FA staining

Cells were seeded on glass coverslips and allowed to settle overnight and then treated with 20 µm Y-27632 or the vehicle control for 20 h before fixing with 3.2% (vol/vol) paraformaldehyde in PBS. Cells were washed with PBS and permeabilized with 1% (vol/vol) Triton X-100 in PBS and blocked with 2% (wt/vol) bovine serum albumin (A2153; MilliporeSigma) and 10% (vol/vol) donkey serum in 0.02% (vol/vol) Tween in PBS. Cells were incubated overnight at 4°C with mouse anti-paxillin antibody (AHO0492; Thermo Fisher Scientific, RRID:AB_2536312) at 1:100. Cells were washed with 0.02% Tween (vol/vol) in PBS and incubated with the secondary antibody donkey anti-mouse Alexa Fluor 488 (A-21202; Thermo Fisher Scientific, RRID:AB_141607) at 1:200, Texas Red-X Phalloidin (T7471; Thermo Fisher Scientific) at 1:500, and DAPI at 1:500 for 1 h at room temperature. Cells were then washed with 0.02% Tween in PBS and then mounted onto a coverslip using mounting solution (H100010; Thermo Fisher Scientific). Single cells were imaged by collecting z-stacks using a Zeiss LSM800 confocal microscope equipped with a 40×/1.1 NA water immersion objective operated by Zen software.

### Metabolic inhibitor experiments

Vcl KO cells were seeded onto 2D glass surfaces and allowed to settle overnight before treatment with either 20 µm oligomycin to inhibit the ATP synthase or 10 µm iodoacetate to inhibit GAPDH in glycolysis, or the vehicle control, DMSO or H_2_O, respectively. Cells were treated for 6 h, and we verified that metabolic differences were present upon treatment (Fig. S3, G and H). For oligomycin, metabolic differences were verified by treating cells with 20 nM TMRM and quantifying signal intensity in the cells after 6 h of oligomycin treatment. For iodoacetate, metabolic differences were confirmed using 0.146 mM 2-NBDG to assess glucose uptake. The TMRM and 2-NBDG images were collected using the Zeiss LSM800 confocal microscope operated by Zen software and equipped with a 20×/0.8 NA objective to collect z-stacks. Time-lapse videos with a 5-min duration of ∼4 s between frames were collected using a Zeiss LSM800 confocal microscope operated by Zen software and equipped with a 10×/0.3 NA objective and an environmental chamber maintained at 37°C and 5% CO_2_.

### Rho activation experiments

Parental MDA cells were seeded onto glass surfaces in complete media and allowed to settle overnight before treatment with 50 µm Rho Activator I (CN01; Cytoskeleton) or the vehicle control (DMSO) and 20 nM TMRM. After 1 h of treatment, cells were imaged using a Zeiss LSM800 confocal microscope operated by Zen software and equipped with a 20×/0.8 NA objective and an environmental chamber maintained at 37°C and 5% CO_2_ to collect z-stacks to assess TMRM signal, in addition to utilizing a 10×/0.3 NA objective to collect time-lapse videos to quantify cell blebbing. Time-lapse videos were collected over a 5-min period with ∼4-s intervals between frames.

### Cell spreading rate experiments

Following trypsinization, parental MDA cells were seeded onto tissue culture plates in media containing either 20 µM oligomycin or 10 µM iodoacetate or the vehicle control. We immediately started time-lapse microscopy to monitor the cell spreading over time using the Cytation 5, equipped with an environmental chamber to maintain the samples at 37°C and 5% CO_2_. To compare spreading over time, cells were analyzed every 4 h for a total time of 12 h.

### Statistical analysis

All statistical analysis was performed using GraphPad Prism 10. The spread of the data was tested for normality using the D’Agostino–Pearson omnibus normality test. For comparing two groups, statistical significance was tested using two-tailed Student’s *t* test or a two-tailed Mann–Whitney test for data with non-normal distribution, where appropriate. For comparing more than two groups, the Kruskal–Wallis analysis of variance testing was used followed by post hoc Tukey’s test. Statistical significance was considered with a P-value <0.05. All data represented are pooled from at least three independent replicate studies, with replicate number and sample size listed in each figure caption. Similar numbers of cells were measured for each biological repeat across an experiment.

### Online supplemental material

This manuscript contains three supplemental figures and 10 supplemental videos. Fig. S1 shows representative western blots for all experiments where siRNA or CRISPR/Cas9 gene editing was used. Fig. S2 shows additional characterization of the MDA-MB-231 Vcl KO cell line, including viability and proliferation assessments. Fig. S3 shows verification of cell responses to inhibitors, including the use of Y-27632, Blebb , oligomycin, and iodoacetate. Video 1 shows a representative video of an MDA cell migrating on a 2D surface at 7 frames per second. Video 2 shows a representative video of a Vcl KO cell migrating on a 2D surface at 7 frames per second. Video 3 shows a representative video of an MDA cell moving in a 1.5 mg/ml collagen gel at 7 frames per second. Video 4 shows a representative video of a Vcl KO cell moving in a 1.5 mg/ml collagen gel at 7 frames per second. Video 5 shows a representative video of a Vcl KO cell expressing LifeAct (red) and stained with MemGlow (green) at 3 frames per second. Video 6 shows a representative video of an MDA cell expressing LifeAct (red) and stained with MemGlow (green) at 3 frames per second. Video 7 shows a representative video of MDA cells moving on a 2D surface that are treated with the vehicle control for Y-27632 (deionized water), played at 7 frames per second. Video 8 shows a representative video of Vcl KO cells moving on a 2D surface that are treated with the vehicle control for Y-27632 (deionized water), played at 7 frames per second. Video 9 shows a representative video of MDA cells moving on a 2D surface that are treated with Y-27632 at 7 frames per second. Video 10 shows a representative video of Vcl KO cells moving on a 2D surface that are treated with Y-27632 at 7 frames per second.

## Supplementary Material

SourceData F1 is the source file for Fig. 1.

SourceData FS1 is the source file for Fig. S1.

## Data Availability

The data are available from the corresponding author upon reasonable request.
