# Supplementary figures and images for: Mechanometabolism of cell adhesion: Vinculin regulates bioenergetics via RhoA-ROCK
Source: J Cell Biol. 2026 Jan 20;225(3):e202504025. doi: 10.1083/jcb.202504025 (PMC12818104; doi:10.1083/jcb.202504025)

1A  
(full blot represented in S1A)

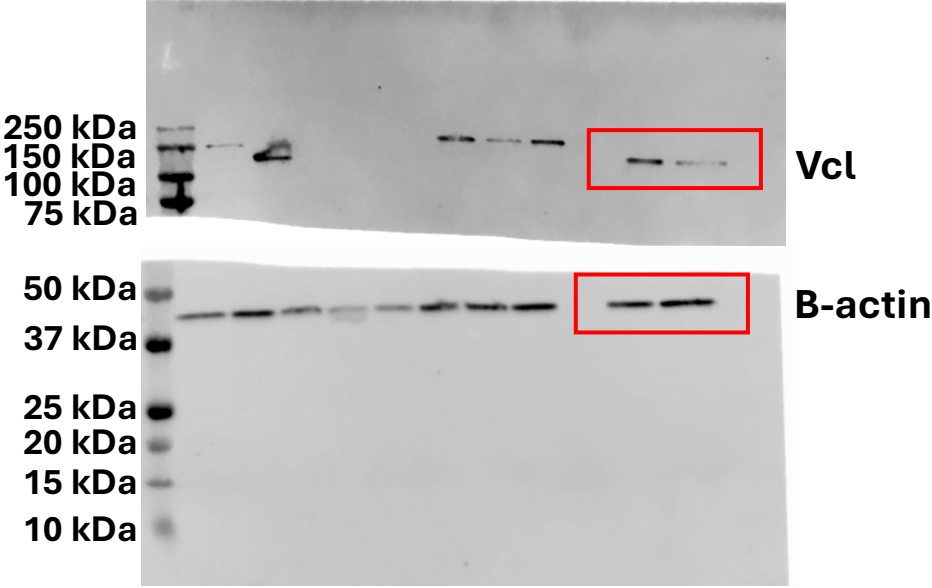

Supplement: SourceData F1 — is the source file for Fig. 1. [file jcb_202504025_sourcedataf1.pdf]

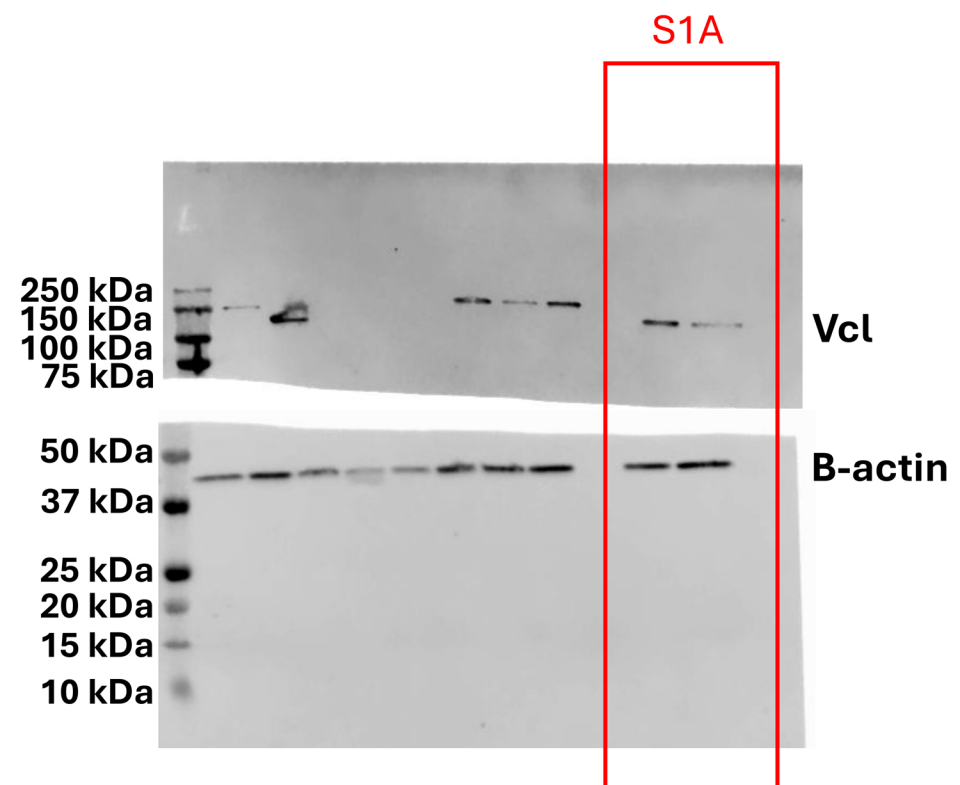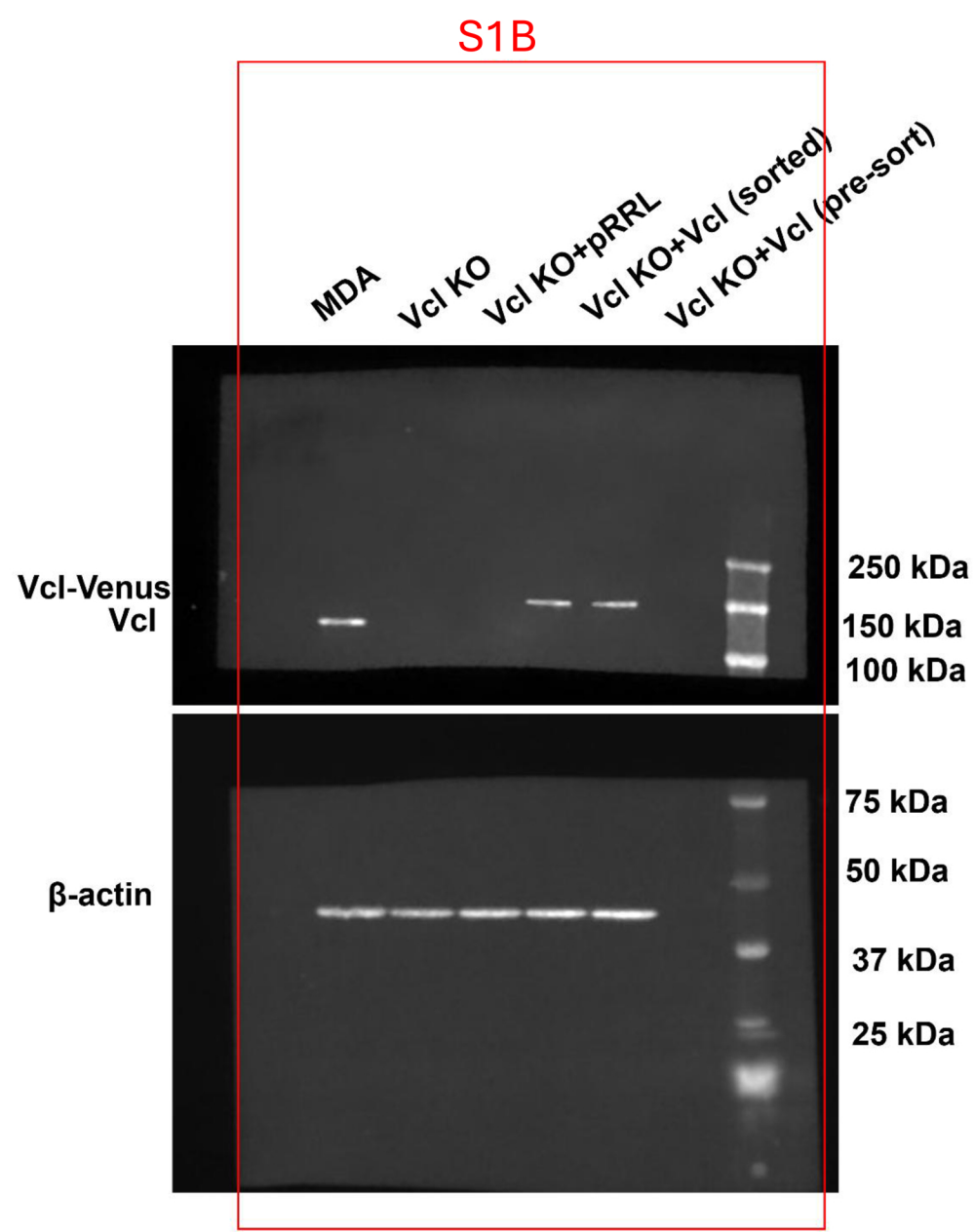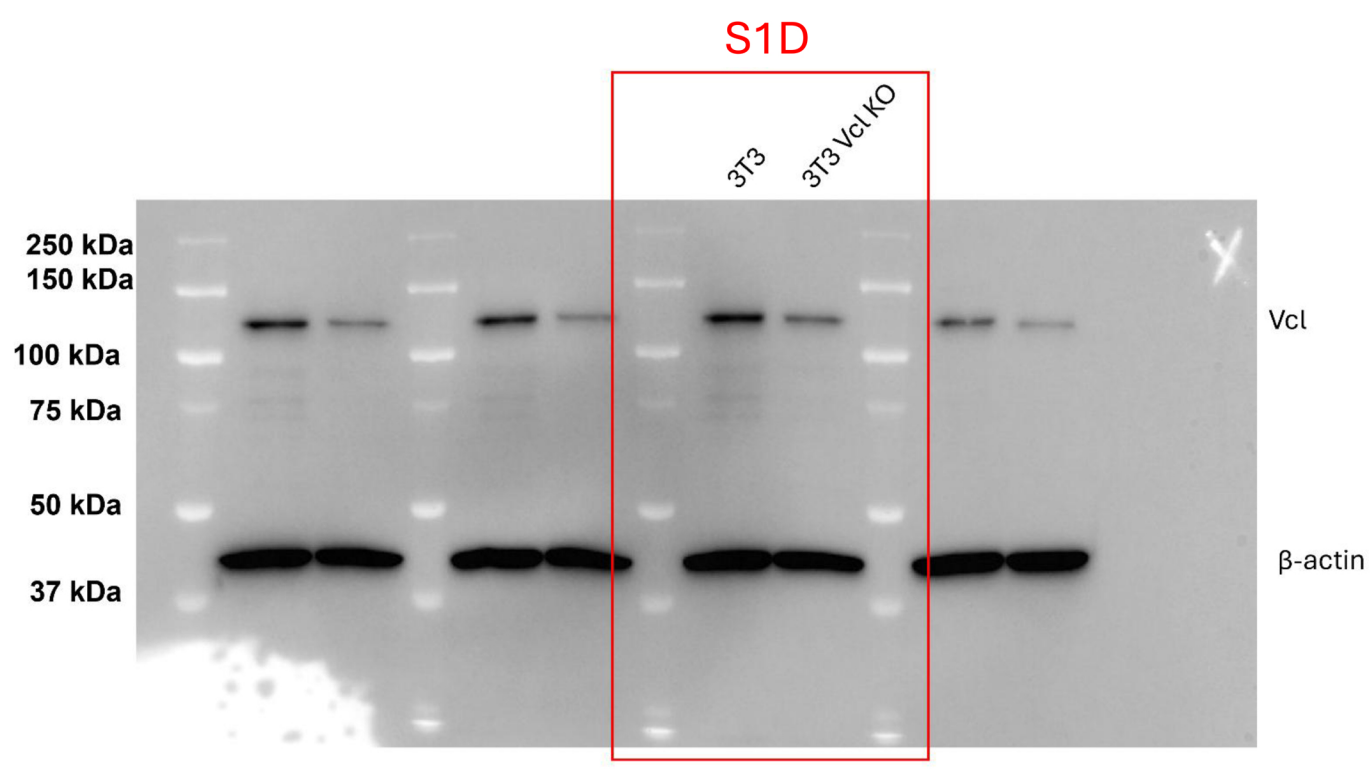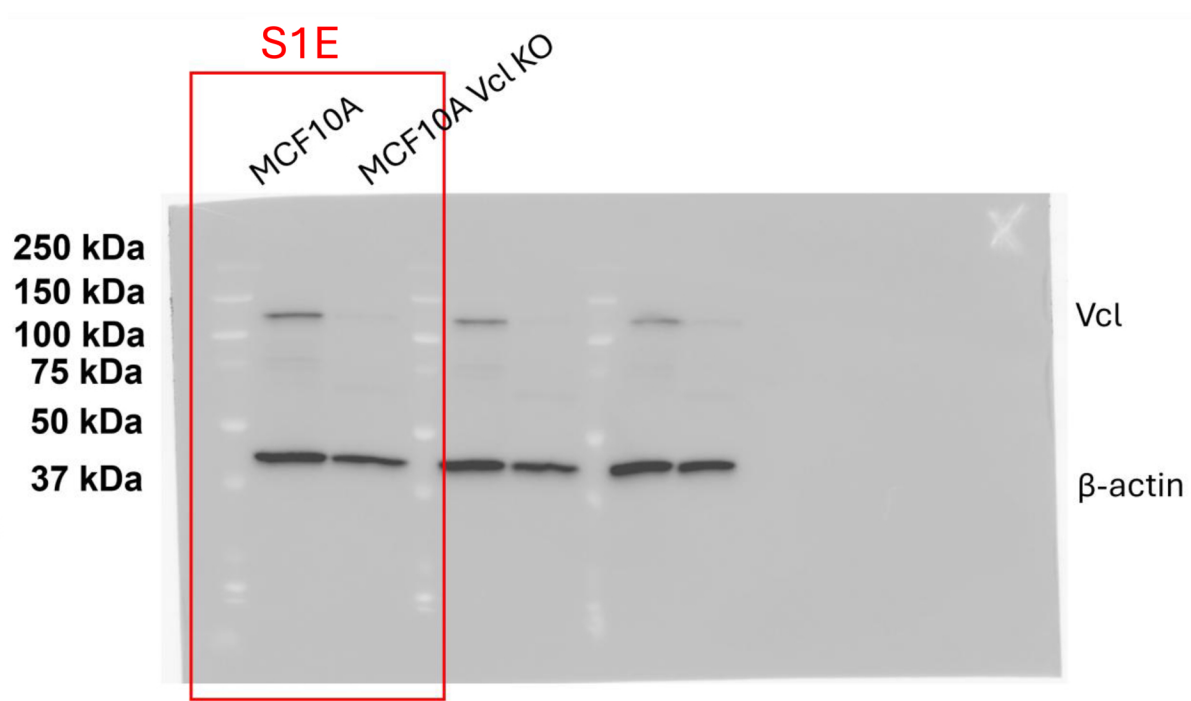

Supplement: SourceData FS1 — is the source file for Fig. S1. [file jcb_202504025_sourcedatafs1.pdf]
